# Ehrlichia chaffeensis TRP120 Is a Wnt Ligand Mimetic That Interacts with Wnt Receptors and Contains a Novel Repetitive Short Linear Motif That Activates Wnt Signaling

**DOI:** 10.1128/mSphere.00216-21

**Published:** 2021-04-21

**Authors:** Madison R. Rogan, LaNisha L. Patterson, Caitlan D. Byerly, Tian Luo, Slobodan Paessler, Veljko Veljkovic, Bethany Quade, Jere W. McBride

**Affiliations:** a Department of Pathology, University of Texas Medical Branch, Galveston, Texas, USA; b Department of Microbiology & Immunology, University of Texas Medical Branch, Galveston, Texas, USA; c Center for Biodefense and Emerging Infectious Diseases, University of Texas Medical Branch, Galveston, Texas, USA; d Sealy Institute for Vaccine Sciences, University of Texas Medical Branch, Galveston, Texas, USA; e Institute for Human Infections and Immunity, University of Texas Medical Branch, Galveston, Texas, USA; f BiomedProtection, LLC, Galveston, Texas, USA; University of Kentucky

**Keywords:** *Ehrlichia chaffeensis*, SLiM, TRP120, Wnt ligand, tandem repeat

## Abstract

Ehrlichia chaffeensis expresses the TRP120 multifunctional effector, which is known to play a role in phagocytic entry, on the surface of infectious dense-cored ehrlichiae, but a cognate host receptor has not been identified. We recently reported that *E. chaffeensis* activates canonical Wnt signaling in monocytes to promote bacterial uptake and intracellular survival and that TRP120 was involved in this activation event. To identify the specific mechanism of pathway activation, we hypothesized that TRP120 is a Wnt signaling ligand mimetic that initiates Wnt pathway activity through direct interaction with the Wnt pathway Frizzled family of receptors. In this study, we used confocal immunofluorescence microscopy to demonstrate very strong colocalization between *E. chaffeensis* and Fzd2, 4, 5, 7, and 9 as well as coreceptor LRP5 at 1 to 3 h postinfection. Direct binding between TRP120 and multiple Fzd receptors was further confirmed by enzyme-linked immunosorbent assay (ELISA) and surface plasmon resonance (SPR). Interfering RNA knockdown of Wnt receptors, coreceptors, and signaling pathway components significantly reduced *E. chaffeensis* infection, demonstrating that complex and redundant interactions are involved in Wnt pathway exploitation. We utilized *in silico* approaches to identify a repetitive short linear motif (SLiM) in TRP120 that is homologous to Wnt ligands and used mutant SLiM peptides and an α-TRP120-Wnt-SLiM antibody to demonstrate that the TRP120 Wnt SLiM activates the canonical Wnt pathway and promotes *E. chaffeensis* infection. This study reports the first example of bacterial mimicry of Wnt pathway ligands and highlights a pathogenic mechanism with potential for targeting by antimicrobial therapeutics.

**IMPORTANCE** Upon infecting mammalian hosts, Ehrlichia chaffeensis establishes a replicative niche in microbe-eating immune system cells where it expertly orchestrates infection and spread. One of the ways *Ehrlichia* survives within these phagocytes is by activating evolutionarily conserved signaling pathways including the Wnt pathway; however, the molecular details of pathway hijacking have not been defined. This study is significant because it identifies an ehrlichial protein that directly interacts with components of the Wnt receptor complex, influencing pathway activity and promoting infection. Consequentially, *Ehrlichia* serves as a unique tool to investigate the intricacies of how pathogens repurpose human immune cell signaling and provides an opportunity to better understand many cellular processes in health and disease. Furthermore, understanding how this bacterium utilizes its small genome to survive within cells that evolved to destroy pathogens will facilitate the development of antibacterial therapeutics that could target *Ehrlichia* as well as other intracellular agents of human disease.

## INTRODUCTION

Ehrlichia chaffeensis is a Gram-negative, obligately intracellular bacterium and causative agent of the most prevalent life-threatening, tick-borne disease in the United States, human monocytic ehrlichiosis (HME) (1, 2). *E. chaffeensis* primarily infects monocytes and forms microcolonies within cytosolic, membrane-bound vacuoles (1). Entry of the bacterium into the host cell involves binding of the *E. chaffeensis* outer membrane protein EtpE to the lipid raft-localized receptor DNase X (3). DNase X^−/−^ murine peripheral blood mononuclear cells can be infected with *E. chaffeensis* and display reduction in infection by about 50%, suggesting other signaling events are at play that facilitate bacterial attachment to and entry into the monocyte (3–6). Furthermore, as DNase X is not a transmembrane protein, other membrane proteins are likely involved in order to transduce a phagocytosis signal into the host cytosol.

*E. chaffeensis* expresses tandem repeat protein (TRP) effectors, including TRP32, TRP47, TRP75, and TRP120, which are known to modulate the host cell through binding of host DNA and various host proteins (7–17). These four TRPs are also present on the surface of the infectious dense-cored ehrlichiae, implicating a role for these proteins in invasion of the host cell (10, 18–20). Previous studies have defined TRP120 as an *E. chaffeensis* adhesin that stimulates bacterial entry (20, 21). Furthermore, microspheres coated with recombinant TRP120 can stimulate uptake by human monocytes, which can be blocked with a small-molecule inhibitor of the Wnt signaling pathway (22). This suggests that Wnt signaling and TRP120 play a role in cell invasion by *E. chaffeensis*, but the domains responsible for TRP120 ligand activity and host receptors involved in activation of Wnt signaling by *E. chaffeensis* are not defined.

Wnt signaling is a conserved eukaryotic signal cascade comprising canonical and noncanonical pathways that regulate events including cell fate, development, and cell polarity, as well as innate immunity-associated events such as autophagy, cytokine expression, and phagocytosis (23–28). Dysregulation of the pathway has been widely studied in multiple cancer types, but growing research within the past decade has defined the pathway as a target of pathogenic bacterial virulence strategies (29). Under normal physiological conditions, the signal cascade is activated through the binding of one of 19 secreted Wnt ligands to one of 10 Fzd receptors and a cognate coreceptor present on the surface of the signal-receiving cell. Depending on the combination of binding partners in this Wnt receptor complex, the signal is transduced to the intracellular mediator Disheveled (Dvl) and further amplified through either canonical or noncanonical pathways. Canonical Wnt signaling inhibits the β-catenin destruction complex consisting of adenomatous polyposis coli (APC), Axin, casein kinase (CK), and glycogen synthase kinase 3 beta (GSKβ), allowing β-catenin to evade proteasomal degradation, translocate into the nucleus, and activate transcription of canonical Wnt pathway target genes including cell cycle progression and cell motility genes. In the noncanonical pathways, Dvl signals for the opening of intracellular Ca^2+^ stores, which activates phospholipase C (PLC) and nuclear factor of activated T cells (NFAT) target gene transcription, or signals through small GTPases to regulate actin polymerization and control cell motility and polarization (30, 31).

We have previously published that inhibition of Wnt signaling blocks ehrlichial entry, indicating *E. chaffeensis* effectively establishes infection through activation of Wnt signaling (22). However, how the pathway is activated during infection is unknown. Partial loss of Wnt pathway receptor Fzd5 or Fzd9 through RNA-mediated gene silencing significantly reduces infection, indicating activation of the pathway during infection occurs at the level of the receptor complex (22). Whether TRP120 expressed on the surface of the bacterium acts as an adhesin by directly engaging a Wnt pathway receptor remains to be discovered.

The Wnt signaling pathway has been found to be largely controlled by short linear motifs (SLiMs) within key pathway components (32). SLiMs are amino acid sequences typically found within intrinsically disordered protein domains that confer context-specific functionality when a defined tertiary structure is lacking (33). In the Wnt signaling pathway, SLiMs dictate protein-protein interactions necessary for Wnt signal transduction. Considerable research has demonstrated that pathogenic viruses and bacteria hijack host cells through molecular mimicry of eukaryotic SLiMs, which enables interference with various cell processes (34). Indeed, we have shown that TRP120, an intrinsically disordered protein, manipulates the host cell through hijacking posttranslational modification pathways, interacting with host DNA and proteins, and enzymatic ubiquitin ligase activity (15, 35–38). TRP120 possesses at least 45 predicted unique SLiMs with 181 instances as identified by the Eukaryotic Linear Motif database, some of which have been experimentally verified such as possession of a HECT E3 ubiquitin ligase domain in the C-terminal and SUMOylation motifs throughout the protein (36, 37, 39). Molecular mimicry of Wnt ligands through possession of eukaryotic SLiMs may be a mechanism by which TRP120 hijacks Wnt pathway activity for bacterial infection and survival. Such Wnt manipulation mechanisms have not yet been described for any pathogenic virus or bacterium.

In this study, we identify a mechanism for *E. chaffeensis* activation of Wnt signaling involving TRP120 interaction with the Wnt signaling pathway receptor complex. TRP120 interacts with Fzd homologues and contains a SLiM which molecularly mimics endogenous Wnt ligands. This Wnt SLiM mimic is necessary and sufficient for activation of Wnt/β-catenin canonical signaling in host monocytes and facilitates *E. chaffeensis* infection. TRP120-Fzd receptor engagement provides a mechanism for activation of β-catenin nuclear localization and overall pathway activity observed during infection. Because of the role of the Wnt pathways in regulating innate immunity, phagocytosis, and autolysosome biogenesis, understanding the mechanism through which *E. chaffeensis* activates the pathway provides deeper insight into how the bacterium hijacks conserved cellular events within its host to establish and maintain infection. Furthermore, this *E. chaffeensis* entry-related mechanism supplements the current model of host cell invasion by identifying signaling events that transduce from extracellular bacterial attachment to an intracellular signal.

## RESULTS

### *E. chaffeensis* colocalizes with Fzd homologues during infection.

**FIG 1:**
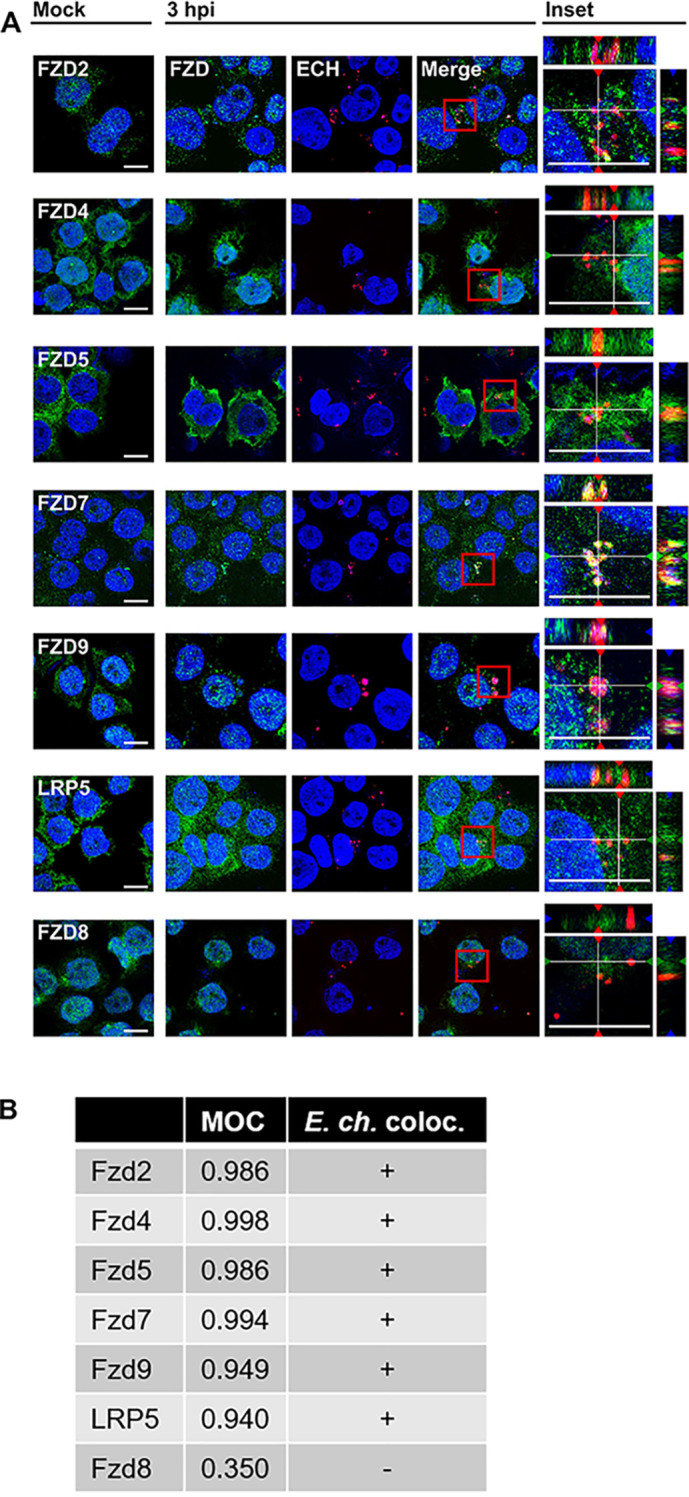
*E. chaffeensis* colocalizes with Wnt receptor complex proteins early in infection. (A) *E. chaffeensis* colocalizes with Fzd2, 4, 5, 7, and 9 and LRP5 but not Fzd8. *E. chaffeensis*-infected (MOI 200) or mock-infected THP-1 cells were harvested 3 hpi, dual stained for *E. chaffeensis* (red) and respective Fzd or LRP antibodies (green), and visualized by confocal fluorescence microscopy. Nuclei are stained with DAPI (blue). Images are single 0.3-μm slices through the respective samples. Gridlines through merged image on far right represent stacked slices through Z plane visualized in the corresponding XZ and YZ orthogonal projections in order to demonstrate colocalization of red and green channels. Red box indicates inset of merged image. Scale bar = 10 μm. Images are representative of three independent experiments. (B) Table depicting positive or negative colocalization of *E. chaffeensis* and respective Fzd or LRP homologues within respective fields as determined by Mander’s overlap coefficient (MOC). Positive (“+”) colocalization was determined as an MOC of 0.6 or higher.

To understand the mechanism of *E. chaffeensis* activation of Wnt signaling in monocytes early during infection, we investigated the role of Fzd receptors and LRP coreceptors during *E. chaffeensis* host cell entry and infection. We first determined that all 10 Fzd homologues and 2 LRP homologues are expressed in THP-1 cells by reverse transcription-quantitative PCR (RT-qPCR) of mRNA (data not shown). *E. chaffeensis* has been reported to bind and trigger entry into the host cell within 3 h, so we next identified which Wnt pathway receptors and coreceptors, if any, colocalize with *E. chaffeensis* during this phase of infection, which could suggest if any are utilized as a receptor for attachment or entry (40). We harvested THP-1 cells infected with *E. chaffeensis* at 3 h postinfection (hpi) and dual stained for Fzd or LRP homologues and *E. chaffeensis* and visualized samples by confocal immunofluorescence microscopy. *E. chaffeensis* and Fzd2, 4, 5, 7, and 9 and LRP5 strongly colocalized at this time point, while weak colocalization was detected between *E. chaffeensis* and Fzd8, which is included as a representative image of negative colocalization (Fig. 1A). Fzd1, 3, 6, and 10, and LRP6, also did not colocalize with *E. chaffeensis* (Fig. 1A and see Fig. S1A in the supplemental material). Of note, there were no apparent differences in the subcellular distributions of each Fzd homologue between mock- and *E. chaffeensis*-infected cells. We quantified colocalization using Mander’s overlap coefficient (MOC), which provides a value (0 to 1) corresponding to the strength of two channels’ fluorescence intensities overlapping at corresponding pixels (41). *E. chaffeensis* very strongly (0.8 to 1.0) colocalized with the respective Fzd and LRP homologues (Fig. 1B). To determine if colocalization with monocyte-expressed Fzd receptors is specific to *E. chaffeensis*, we infected THP-1 cells with the Gram-negative bacterium Rickettsia parkeri and assessed colocalization with Fzd5 after bacterial attachment. *R. parkeri* demonstrated weak colocalization with Fzd5 (Fig. S1B). These data suggest multiple Fzds are directly interacting with *E. chaffeensis* as it enters the cells, indicating this family of transmembrane proteins may be a novel receptor on the monocyte for the bacterium and a point of activation for Wnt pathway activity that has been observed during *E. chaffeensis* infection.

10.1128/mSphere.00216-21.1 FIG S1 *E. chaffeensis* and *R. parkeri* do not colocalize with some Wnt receptor complex proteins early in infection. (A) *E. chaffeensis* does not colocalize with Fzd1, 3, 6, and 10 and LRP6. Mock- or *E. chaffeensis-*infected (MOI 200) THP-1 cells were harvested 3 hpi, dual stained for *E. chaffeensis* (red) and respective Fzd/LRP antibodies (green), and examined by confocal fluorescence microscopy. Nucleus are stained with DAPI (blue). Red box indicates inset. Scale bar = 10 μm. Images are representative of three independent experiments. (B) THP-1 cells were infected with Rickettsia parkeri, and cells were centrifuged at 2,000 rpm for 5 min to facilitate attachment of bacteria to cells. THP-1 cells were then harvested and dual stained for *R. parkeri* (red) and Fzd5 (green). Nuclei are stained with DAPI (blue). Red box indicates inset. Scale bar = 10 μm. Images are representative of three independent experiments. Download FIG S1, JPG file, 2.0 MB. Copyright © 2021 Rogan et al. 2021 Rogan et al. https://creativecommons.org/licenses/by/4.0/ This content is distributed under the terms of the Creative Commons Attribution 4.0 International license.

### TRP120 directly interacts with Fzd5.

**FIG 2:**
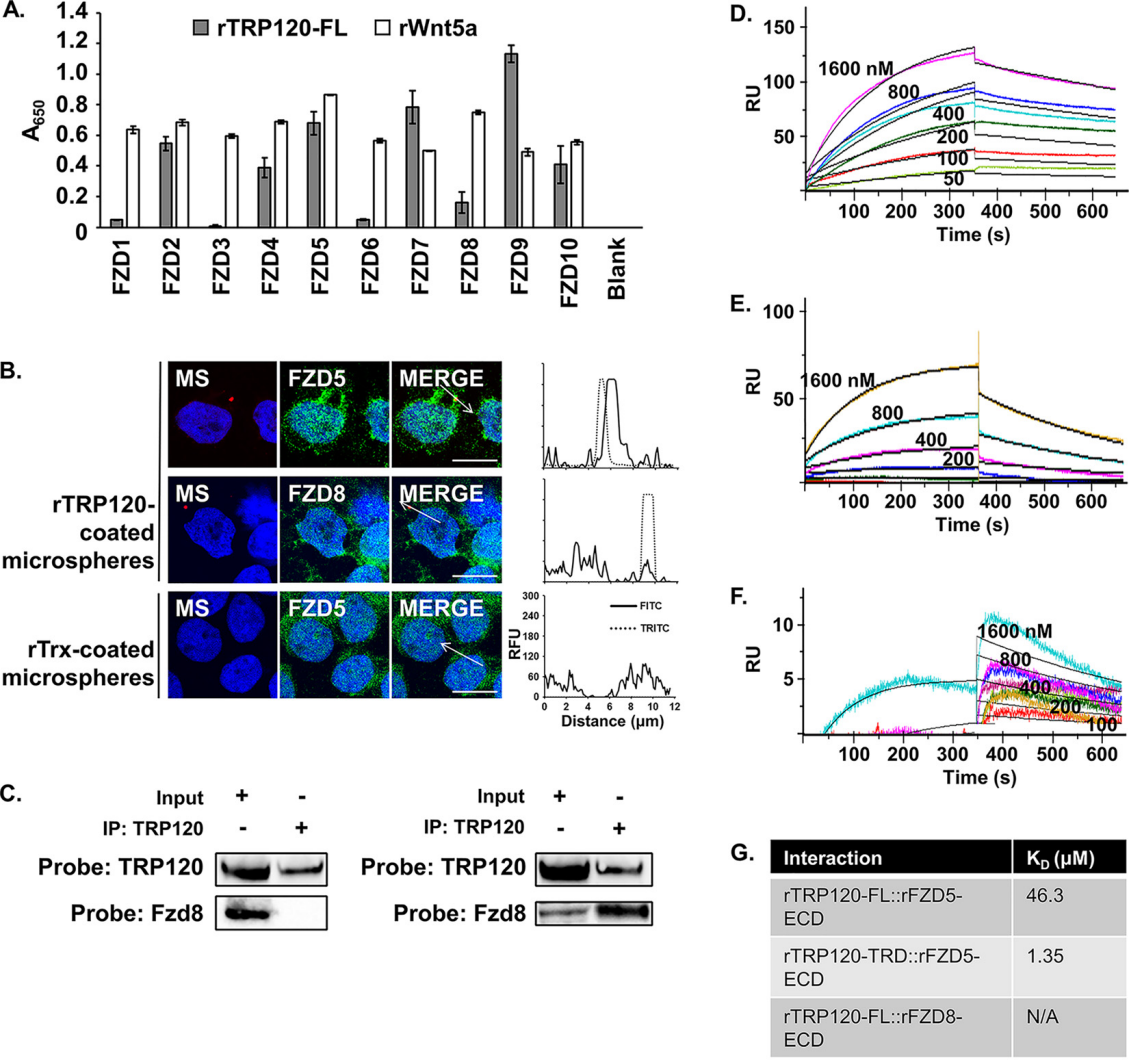
TRP120 directly interacts with Fzd5. (A) rTRP120-FL interacts with rFzd2, 4, 5, 7, 9, and 10 ECD truncate proteins. To measure direct binding of TRP120 and Fzd proteins, recombinant Fzd extracellular domain (ECD) truncated proteins dissolved in PBS, or a blank, were adsorbed onto an ELISA plate and rTRP120-FL (gray) or rWnt5a (white) was added as an interacting protein. Positive interactions were detected by incubation with α-TRP120-I1 or α-Wnt5a followed by alkaline phosphatase-conjugated secondary antibodies. Data are an average from three independent experiments, and values represent the mean plus or minus standard deviation. (B) THP-1 cells were treated with rTrx- or rTRP120-FL-coated microspheres for 1 h. Cells were dual stained for coated microspheres (“MS,” tetramethylrhodamine isothiocyanate [TRITC], red) or Fzd (fluorescein isothiocyanate [FITC], green), and nuclei were stained with DAPI (blue). Arrow represents dimensions of line profile graph of FITC channel (black line) and TRITC channel (dashed line). Scale bar = 10 μm. Images are representative of three independent experiments. (C) THP-1 cells were infected with *E. chaffeensis* (MOI 100), and whole-cell lysate was harvested 70 hpi. Lysate was run over immobilized α-TRP120, and the input lysate as well as the immunoprecipitated eluates was probed for Fzd5 and Fzd8 by immunoblotting. (D to F) One hundred nanomolar rTRP120-FL (D and F) or rTRP120-TRD (E) was immobilized on a Ni-nitrilotriacetic acid (NTA) sensor chip, and 2-fold dilutions (1,600 nM to 50 nM) of solubilized rFzd5-ECD (D and E) or rFzd8-ECD (F) were sequentially run over the chip to determine binding affinity as analyzed by Biacore T100. Sensorgrams are representative of two independent experiments. (G) Binding affinities are reported.

TRP120 is expressed on the surface of the infectious dense-cored (DC) ehrlichiae and mediates both bacterial attachment to host cells and stimulation of phagocytosis (20, 21). However, a cognate receptor on the monocyte has not been identified. Because our previous data demonstrate that TRP120 can trigger phagocytosis through an unknown Wnt-dependent mechanism and that TRP120 is capable of stimulating pathway activity, we wanted to investigate whether TRP120 acts as a Wnt ligand mimic to bind Fzd receptors and activate pathway activity (22, 42). We first performed an enzyme-linked immunosorbent assay (ELISA) in which recombinant, full-length TRP120 (rTRP120-FL) was screened for interaction with immobilized recombinant extracellular domains (ECD) of each Fzd homologue. Recombinant Wnt5a (rWnt5a) was used as a comparison for interaction intensity as Wnt5a is known to interact with multiple Fzds (43–51). rFzd5-ECD, a known receptor of Wnt5a, was found to be a relatively strong interacting partner of rTRP120-FL, as were rFzd2-ECD, 4-ECD, 7-ECD, 9-ECD, and 10-ECD (Fig. 2A). rFzd1-ECD, 3-ECD, 6-ECD, and 8-ECD only marginally interacted. Fzd5 has been shown to participate in a phagocytosis signaling pathway that is active in bacterial infection of macrophages (26), so we chose to further investigate the TRP120-Fzd5 interaction as a model of TRP120 Fzd receptor binding and pathway activation.

To demonstrate if the early colocalization of *E. chaffeensis* and Fzd5 during infection is a binding event dependent on ehrlichial surface-localized TRP120, we coated microspheres in rTRP120-FL, incubated them with THP-1 cells, and determined colocalization with Fzd5 by confocal fluorescence microscopy. rTRP120-FL-coated microspheres bound to THP-1 cells and colocalized with Fzd5 but not Fzd8. Fzd8 served as a negative control because it did not colocalize with *E. chaffeensis* during infection and the recombinant protein weakly interacted with TRP120. rTRP120 is a thioredoxin (Trx)-fusion recombinant protein; therefore, thioredoxin alone purified from an empty expression vector is used as a negative control for rTRP120 protein treatments. rTrx-coated microspheres could neither bind nor colocalize with Fzd5, indicating TRP120 is responsible for attachment of microspheres to THP-1 cells (Fig. 2B). Exposure to rTRP120 induces morphological changes in the monocyte, accounting for the variation in cell shape between rTRP120-coated microsphere-treated cells and THP-1 cells that are untreated or exposed to negative-control microspheres. However, this does not impact cell viability (data not shown). While these data only indicate TRP120 can colocalize with Fzd5 in THP-1 cells, we confirmed direct interaction by immunoprecipitating TRP120 from the lysate of infected THP-1 cells harvested at 70 hpi, the end of an infection cycle when ehrlichiae have transitioned to the TRP120-expressing DC, which ensures that sufficient levels of TRP120 are expressed to detect interactions with host proteins. Fzd5 coimmunoprecipitated with TRP120, while Fzd8 did not (Fig. 2C). This demonstrates that interaction between TRP120 and Fzd5 is occurring during infection of monocytes. We further assessed the direct interaction of rTRP120 and rFzd5-ECD by surface plasmon resonance to determine the binding affinity, with rFzd8-ECD serving as a negative control. The *K_D_* (equilibrium dissociation constant) for rTRP120-FL and rFzd5-ECD interaction was found to be 46.3 μM (Fig. 2D), and the rTRP120-tandem repeat domain truncate construct (rTRP120-TRD) interacted with rFzd5-ECD with a *K_D_* of 1.35 μM (Fig. 2E). rFzd8-ECD was again used as a negative control (Fig. 2F). The *K_D_* of each interaction is compiled in Fig. 2G. These data demonstrate the role of Fzd5 as a receptor for the ehrlichial surface protein TRP120 in host monocytes and support the idea that the TRP120 TRD may contain a motif that mediates this binding event.

### Components of the Wnt receptor complex are necessary for full *E. chaffeensis* infection.

**FIG 3:**
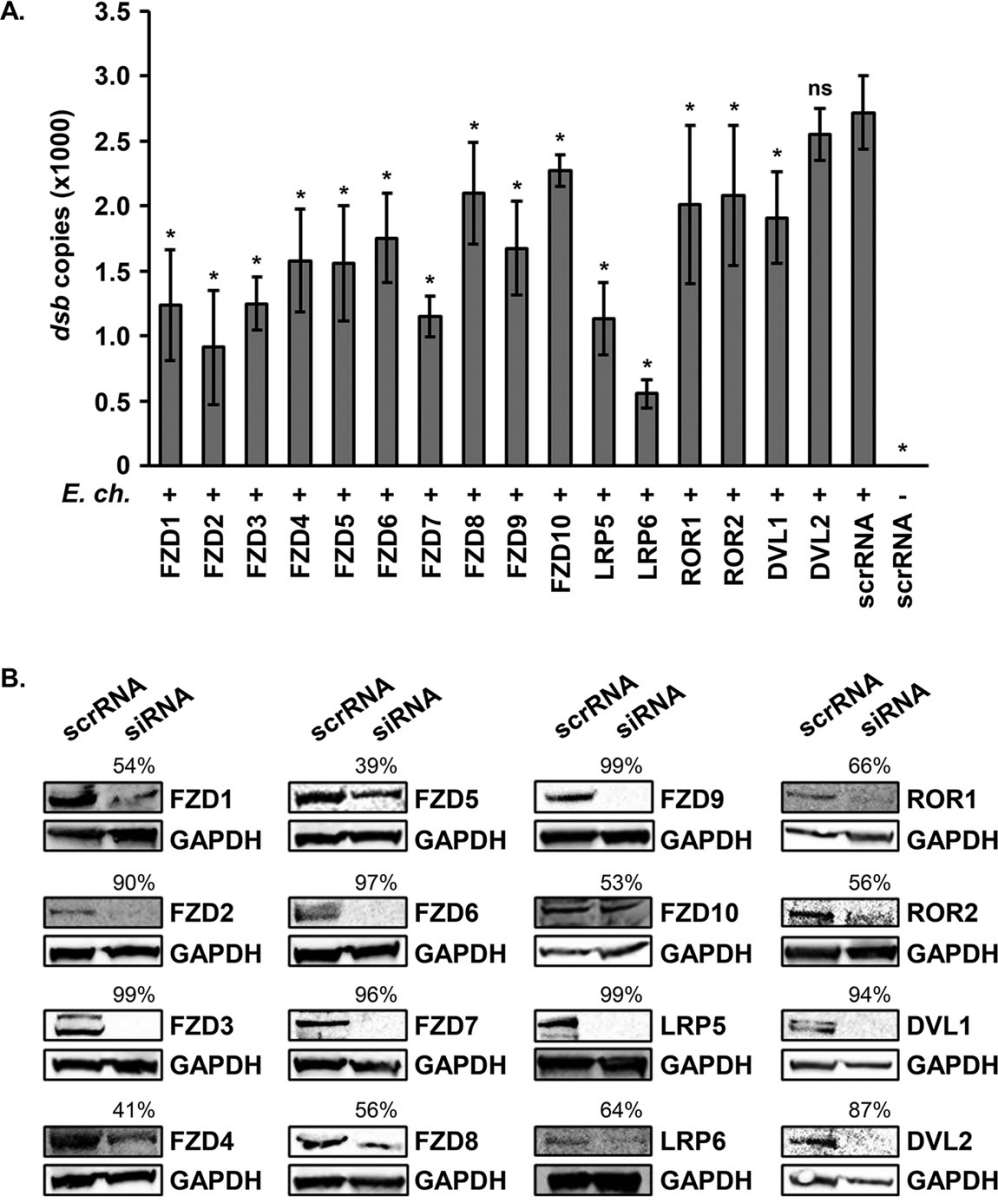
Small interfering RNA silencing of Wnt pathway receptor complex proteins significantly reduces *E. chaffeensis* infection. Small interfering RNA-transfected THP-1 cells were infected or mock infected with *E. chaffeensis* (MOI 100, 24 h posttransfection). (A) Infection level was quantified at 24 hpi by qPCR amplification of ehrlichial *dsb* for each siRNA treatment (*y* axis). Scrambled siRNA (scrRNA) was transfected as a negative control. Values represent the mean plus or minus standard deviation. *, *P* < 0.05; ns, *P* > 0.05. Data are an average from at least three independent experiments. (B) Immunoblots depicting protein level of siRNA targets relative to scrRNA transfection from whole-cell lysates harvested 24 h posttransfection. Number above siRNA lane indicates percent knockdown of protein of interest relative to scrRNA transfection, normalized to GAPDH expression.

To determine if Wnt receptor complex proteins—including 10 Fzd homologues, 4 canonical or noncanonical pathway coreceptors, and 2 Dvl homologues—play a role in *E. chaffeensis* infection of the monocyte, we used interfering RNA to individually silence expression of 16 receptor complex proteins in THP-1 cells. At 24 h post-infection of small interfering RNA (siRNA)-transfected cells, *E. chaffeensis* infection level was significantly reduced in nearly all transfection groups, excluding Dvl2 knockdown, relative to the infection level in scrambled siRNA-transfected cells (Fig. 3A). A relatively high knockdown of respective target protein expression in siRNA-transfected cells compared to scrambled siRNA-transfected cells was achieved at 1 day posttransfection, the day cells were infected (Fig. 3B). Two of the most significant reductions in *E. chaffeensis* infection occurred in LRP5- and LRP6-silenced cells. Knockdown of noncanonical pathway coreceptors ROR1 and ROR2 resulted in impaired infection as well, although to a lesser degree. Loss of intracellular signal relay protein Dvl1 significantly reduced infection, while Dvl2 knockdown did not, suggesting redundant mechanisms are at play at this level of the signaling cascade. Altogether, these data suggest that the Wnt receptor complex is directly involved in *E. chaffeensis* infection by serving as a TRP120 receptor.

### TRP120 is a predicted Wnt ligand mimic.

**FIG 4:**
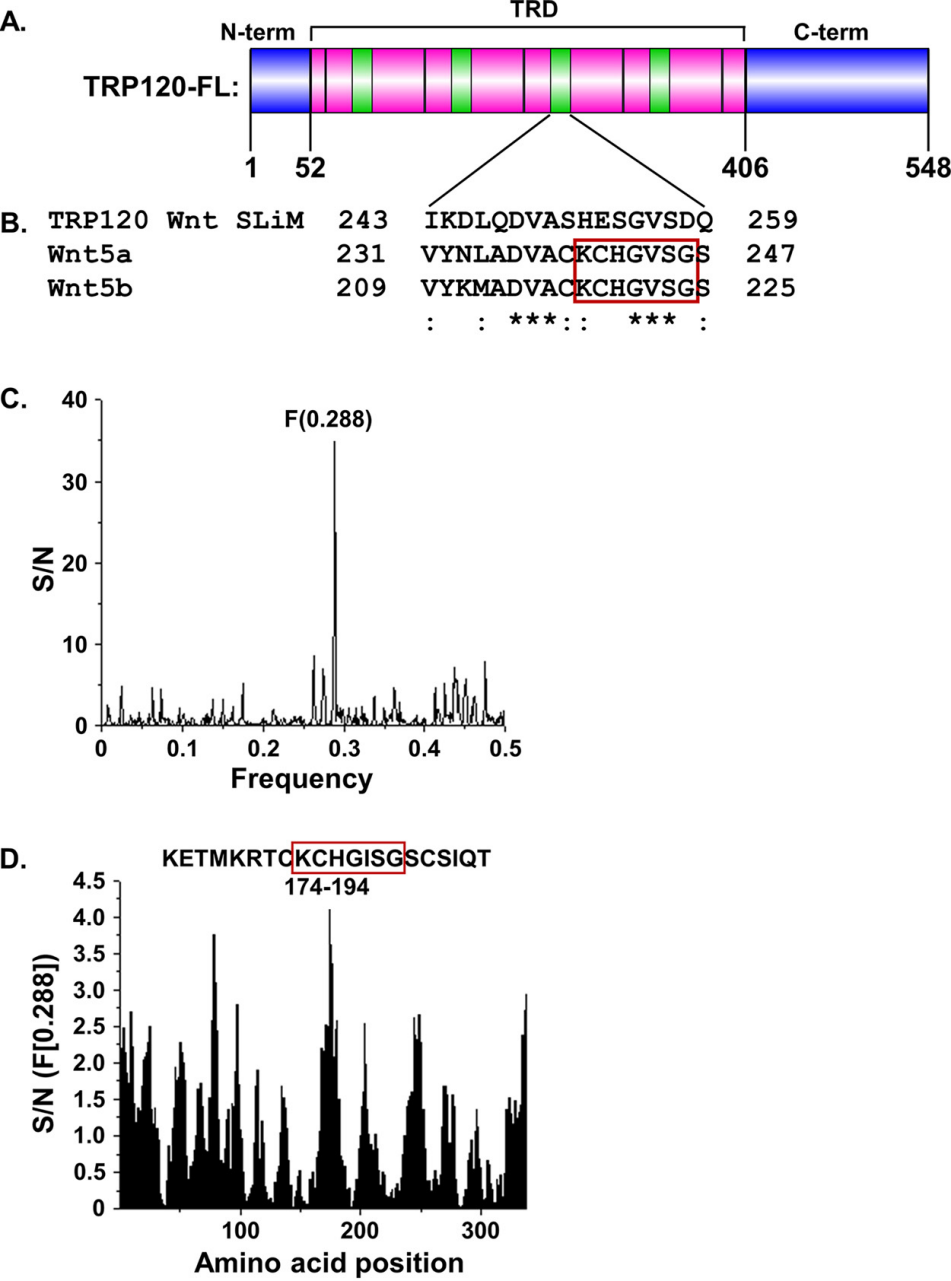
TRP120 is a predicted Wnt ligand mimic. (A) Protein domain map of TRP120 indicating location of the Wnt SLiM (green) repeated throughout tandem repeat domain (TRD, pink). The N terminus and C terminus are indicated in blue. (B) Protein sequence alignment of TRP120 Wnt SLiM and corresponding homologous sequences in Wnt5a and Wnt5b. Fzd-binding site 1 is boxed in red. A colon indicates similar amino acid substitutions, and an asterisk indicates identical amino acids across the three alignments. (C and D) ISM analysis of TRP120 and XWnt8. (C) Cross-spectrum of TRP120 (accession no. AAO12927.1) and XWnt8 (NP_001081637.1) with ISM frequencies (*x* axis) plotted against normalized amplitude for each component (*y* axis). (D) Scanning of the amino acid sequence of XWnt8 along ISM F[0.288] with Fzd-binding site 1 boxed in red.

Following the observation that TRP120 directly interacts with Fzd receptors, we wanted to define the interacting motif within TRP120. Wnt ligands have been shown to directly bind Fzd receptors through the hydrophobic interactions of two distinct motifs with the receptor ECD (52). TRP120 is composed of a tandem repeat domain flanked by the N and C termini. The N terminus, TRD, and C terminus have been shown to possess various functional SLiMs that are relevant to infection of the monocyte, including posttranslational modification motifs, DNA-binding motifs, and ubiquitin ligase catalytic motifs (Fig. 4A). We used NCBI Protein BLAST to investigate if TRP120 shares amino acid sequence similarity with either of the Wnt ligand receptor-binding motifs, which might confer the ability to engage with a Fzd receptor. Significant sequence similarity was found across nearly all Wnt ligands (data not shown), while Wnt5a and Wnt5b both demonstrated significant sequence similarity between one of the receptor-binding motifs and a region within the TRP120-TRD which was able to interact with the Fzd5-ECD (Fig. 4B). This amino acid sequence was termed the TRP120 Wnt SLiM. By nature of the TRD, the Wnt SLiM is repeated throughout the TRD four times.

While a bacterial protein can mimic a eukaryotic motif, amino acid sequence similarity does not necessarily determine functional similarity. To predict functional similarity, we performed an information spectrum method (ISM) analysis comparing TRP120 to Xenopus laevis Wnt8 (XWnt8). ISM is a well-documented tool that identifies shared characteristics among two molecules through predicting similar long-wave frequency vibrations that dictate various protein functions (53). In an ISM cross-spectrum of the electron-ion interaction potentials (EEIP) of TRP120 and XWnt8, a significant peak amplitude at frequency 0.288 was identified, indicating a shared biological function between these two proteins (Fig. 4C). Scanning of the EEIP sequence of XWnt8 along the frequency 0.288 identified that the amino acids contributing to this shared biological function reside within the conserved Fzd-binding site 1 of XWnt8 (Fig. 4D). This is the same conserved site of Wnt5a and Wnt5b to which TRP120 displays homology. Together with the data from our BLAST screen, this suggests TRP120 contains a Wnt SLiM in the TRD that has sequence similarity with the receptor-binding site of Wnt ligands and is predicted by ISM to have functional similarity to Wnt ligands.

### TRP120 can induce Wnt/β-catenin pathway activity.

**FIG 5:**
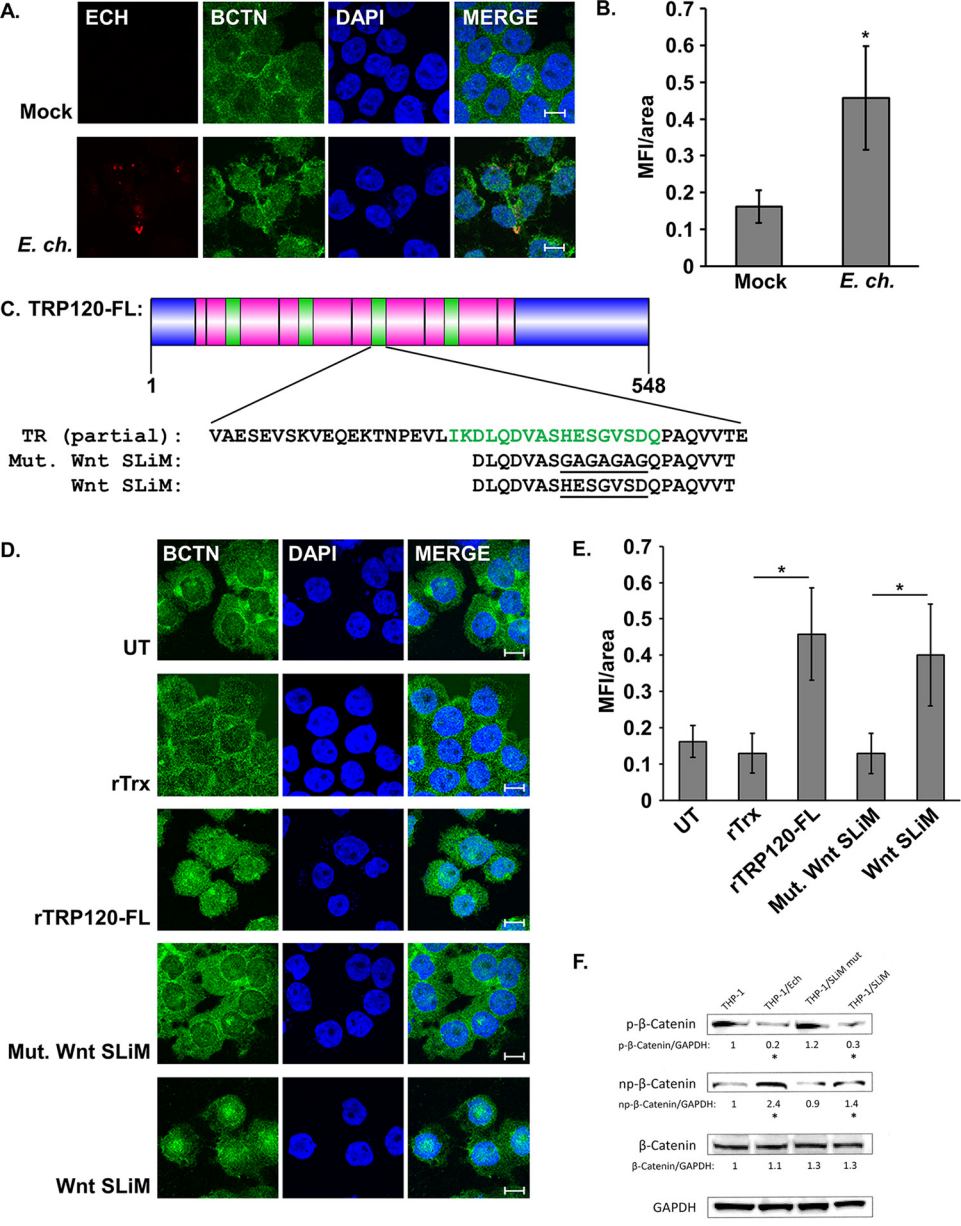
TRP120 activated canonical Wnt signaling through a Wnt SLiM. (A) *E. chaffeensis* infection stimulates β-catenin nuclear translocation. THP-1 cells were infected or mock infected with *E. chaffeensis* (MOI 100), harvested 3 hpi, immunostained for *E. chaffeensis* (red) and β-catenin (BCTN, green), and visualized by confocal fluorescence microscopy. Scale bar = 10 μm. (B) Wnt pathway activation was quantified by measuring mean nuclear fluorescence intensity per area (MFI/area) of β-catenin in infected versus mock-infected cells. (C) Protein domain map of TRP120-FL. The Wnt SLiM is indicated in green. Sequences of peptides used to investigate Wnt pathway activation are aligned with respective region in the TRD (pink). The N terminus and C terminus are indicated in blue. (D) rTRP120-FL and a Wnt SLiM peptide stimulate β-catenin activation. THP-1 cells were treated with indicated recombinant proteins or synthetic peptides (2 μg/ml) for 3 h, immunostained for β-catenin (green), and visualized by confocal fluorescence microscopy. Scale bar = 10 μm. (E) Quantification of Wnt pathway activation by different treatment groups was measured as described above. Values are an average from three independent experiments plus or minus standard deviation. *, *P* < 0.05. (F) Western immunoblot demonstrating decreased phosphorylation of β-catenin in response to *E. chaffeensis* or Wnt SLiM peptide at 3 h postincubation compared to uninfected THP-1 cells and cells incubated with mutant Wnt SLiM peptide. Densitometry values are shown below each respective band and are shown as fold change normalized to GAPDH. *, *P* < 0.05. Significance compared to uninfected control was calculated from three individual experiments (*n* = 3).

After identifying a predicted Wnt SLiM mimicry motif in TRP120-TRD, we asked whether TRP120 can activate the canonical Wnt signaling pathway. We utilized a Wnt/β-catenin activation assay in which activation of Wnt signaling in THP-1 cells was measured by the nuclear translocation of β-catenin via confocal fluorescence microscopy. We first established that *E. chaffeensis* stimulates β-catenin nuclear translocation, which we have previously demonstrated (Fig. 5A and B) (22). We next investigated if rTRP120-FL and a 21-mer peptide containing the Wnt SLiM alone are capable of activating signaling (Fig. 5C). THP-1 cells were treated with solubilized rTRP120-FL or the Wnt SLiM peptide for 3 h, and signaling was measured as described. Both treatments elicited a significant increase in pathway activity, while rTrx as a protein treatment control could not activate the pathway, nor could a mutated Wnt SLiM peptide (Wnt SLiM peptide mut.) (Fig. 5D and E). *E. chaffeensis* and Wnt SLiM peptide both caused decreases in p-β-catenin and increased non-p-β-catenin at 3 h postinfection/treatment (Fig. 5F), and *E. chaffeensis* and Wnt SLiM peptide exhibited similar activation of a subset of Wnt target genes at 12 h postinfection/treatment including BIRC5, BMP4, SOX9, AXIN2, VEGF, FN1, and others (Table S2). These data demonstrate that the TRP120 Wnt SLiM is necessary for activation of Wnt/β-catenin signaling in THP-1 cells and suggest that this ligand mimicry conferred by the Wnt SLiM is responsible for activation of the pathway in the early phase of *E. chaffeensis* infection.

10.1128/mSphere.00216-21.3 TABLE S2 Wnt target gene qPCR analysis. Download Table S2, DOCX file, 0.03 MB. Copyright © 2021 Rogan et al. 2021 Rogan et al. https://creativecommons.org/licenses/by/4.0/ This content is distributed under the terms of the Creative Commons Attribution 4.0 International license.

### Inhibition of the TRP120 Wnt SLiM inhibits Wnt pathway activation and infection.

**FIG 6:**
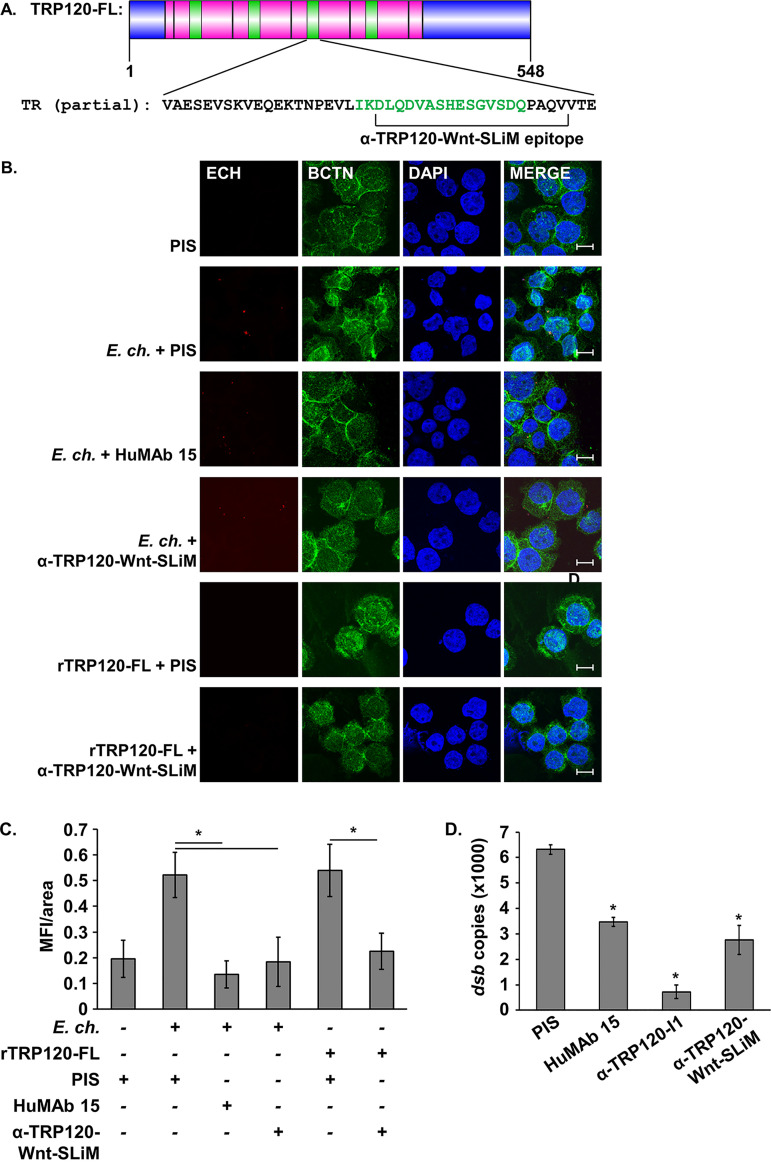
Inhibition of the TRP120 Wnt SLiM prevents activation of canonical Wnt signaling and reduces infection. (A) Protein domain map of TRP120 indicating the overlap of the epitope of α-TRP120-Wnt-SLiM antibody and the TRP120 Wnt SLiM (green) in the tandem repeat domain (pink). The N terminus and C terminus are indicated in blue. (B) Inhibition of the TRP120 Wnt SLiM with α-TRP120-Wnt-SLiM inhibits activation of Wnt signaling by *E. chaffeensis* and rTRP120-FL. THP-1 cells were pretreated with rabbit preimmune serum (PIS), HuMAb 15, or α-TRP120-Wnt-SLiM, subsequently infected (MOI 100) or treated with rTRP120-FL (2 μg/ml), harvested 3 h after treatment, immunostained for *E. chaffeensis* (red) and β-catenin (BCTN, green), and visualized by confocal fluorescence microscopy. Scale bar = 10 μm. (C) Wnt pathway activation was quantified by measuring mean nuclear fluorescence intensity per area (MFI/area) of β-catenin in treated versus untreated cells. (D) THP-1 cells were pretreated with indicated antibodies and infected with *E. chaffeensis* (MOI 100), and infection levels were quantified 24 hpi by qPCR amplification of ehrlichial *dsb* gene. All values are an average from three independent experiments plus or minus standard deviation. *, *P* < 0.05.

To understand the role of the TRP120 Wnt SLiM during *E. chaffeensis* infection, we investigated if blocking of the Wnt SLiM can inhibit Wnt pathway activation by *E. chaffeensis* and reduce *E. chaffeensis* infection level. TRP120-Wnt-SLiM rabbit antiserum was produced by immunization with the Wnt SLiM peptide (Fig. 6A). We then used a neutralization assay to determine how the antibody affects activation of Wnt/β-catenin signaling by *E. chaffeensis*. THP-1 cells were incubated with either a human monoclonal antibody targeting an *E. chaffeensis* outer membrane protein (HuMAb 15) which is known to inhibit infection (54), a negative control of rabbit preimmune serum (PIS), or α-TRP120-Wnt-SLiM antiserum and the cells were subsequently infected for 3 h, after which cells were assayed for β-catenin nuclear translocation. Infection in the presence of HuMAb 15 as well α-TRP120-Wnt-SLiM demonstrated significant reduction in activation of pathway activity relative to the PIS-treated group (Fig. 6B and C). Pathway activity stimulated by rTRP120-FL could also be significantly reduced in the presence of α-TRP120-Wnt-SLiM (Fig. 6B and C) relative to treatment with PIS. These data demonstrate the relevance of the Wnt SLiM of TRP120 to activation of Wnt/β-catenin signaling by *E. chaffeensis*.

We also evaluated inhibition of infection by α-TRP120-Wnt-SLiM compared to HuMAb 15 and α-TRP120-I1, which targets a region of the TRD that is upstream of the Wnt SLiM and has been previously established as neutralizing (55). When THP-1 cells were individually pretreated with these antibodies and subsequently infected, infection was significantly reduced by 24 hpi relative to pretreatment with rabbit preimmune serum, with α-TRP120-Wnt-SLiM achieving infection reduction levels similar to HuMAb 15 (Fig. 6D). This suggests that activation of Wnt signaling by the Wnt SLiM is necessary for enhancement of *E. chaffeensis* infection in monocytes and that this mechanism could be exploited for antimicrobial therapeutics.

## DISCUSSION

Ehrlichial TRP effectors and surface proteins drive the immunoevasion program during infection that enables the obligate intracellular bacterium to survive within the host monocyte. TRP32, TRP47, and TRP120 have been shown to hijack Notch and Wnt signaling pathways to inhibit antibacterial strategies including autophagy and Toll-like receptor (TLR) signaling (42, 56). TRP120 is secreted into the host cell through a type I secretion system and localizes to the cytoplasm and nucleus, and yeast-two-hybrid screening and chromatin immunoprecipitation studies have identified a multitude of TRP120-host molecular interactions within the host cell (15, 16). However, the effect of surface-localized TRP120 on the host cell during *E. chaffeensis* attachment and phagocytosis and the mechanisms involved have not been thoroughly studied.

TRP120 has previously described adhesin and Wnt pathway activating properties. When ectopically expressed in noninvasive Escherichia coli, TRP120 localizes to the surface and triggers phagocytosis of the bacteria by HeLa cells (20). Furthermore, depletion of TRP120 from the surface of *E. chaffeensis* through protease treatment prior to infection of THP-1 cells significantly reduces the number of internalized bacteria at 2 hpi (21). Additionally, we have reported that microspheres coated in rTRP120-FL can be internalized by THP-1 cells, but internalization is inhibited when cells are treated with a Wnt signaling pathway inhibitor. This suggests that the role TRP120 plays in attachment of *E. chaffeensis* to host cells and facilitation of infection may be related to the ability of *E. chaffeensis* to stimulate Wnt pathway activity, but lack of identification of surface receptors relevant to these events has prevented definition of such mechanisms.

**FIG 7:**
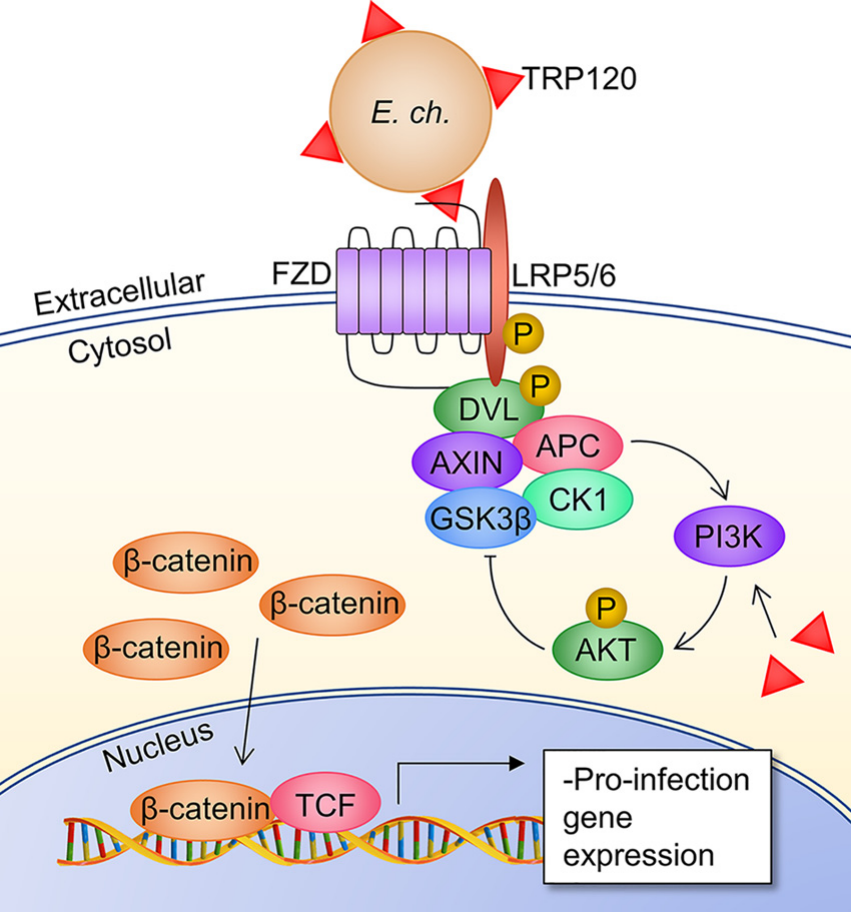
Proposed model of *E. chaffeensis* manipulation of canonical Wnt signaling by TRP120. DC ehrlichia surface-expressed TRP120 directly engages one or more Fzd receptors at the extracellular conserved CRD through a Wnt SLiM repeated in the TRP120 TRD. This results in recruitment of coreceptor LRP5 and activation of canonical Wnt signaling through disassembly of the β-catenin destruction complex (consisting of Axin, APC, GSK3β, and CK1), which allows accumulation of β-catenin in the cytoplasm and its subsequent nuclear translocation for activation of Wnt target genes. These genes are characterized as pro-*E. chaffeensis* infection, as inhibition of signaling impairs bacterial infection (22). Furthermore, TRP120 that is secreted into the host cytoplasm from the *E. chaffeensis* morula during infection drives inhibition of the β-catenin destruction complex through phosphatidylinositol 3-kinase (PI3K)/AKT signaling which amplifies canonical Wnt signaling and promotes infection through suppression of autophagy (42).

Here, we investigated the role of the Wnt receptor complex in *E. chaffeensis* host cell entry and establishment of infection and characterized TRP120 as a Wnt pathway ligand mimic (Fig. 7). There are 10 Fzd homologues in the mammalian genome, and all were found to be expressed by THP-1 cells. At the time of this publication, data describing expression of Fzd family receptors in human peripheral blood mononuclear cell-derived macrophages have been limited to Fzd1, 2, and 5 (57–59), and Fzd5 is the only receptor for which expression has been confirmed in the THP-1 cell line. We confirmed protein-level expression of all 10 homologues in THP-1 monocytes. This not only suggests that canonical and noncanonical Wnt pathways are active in this cell line but also demonstrates a large amount of signaling redundancy in THP-1 cells as the Fzd cysteine-rich domain in the extracellular domain (ECD) in which ligand binding occurs is highly conserved across the family. The colocalization of *E. chaffeensis* with multiple Fzd receptors suggests the bacterium is utilizing this conserved ECD to engage with the receptors. The Clostridium difficile enterotoxin TcdB has been shown to interact with Fzd1, 2, and 7 in the ECD. More specifically, TcdB binds within the conserved receptor domain (CRD) at a binding site distinct from Wnt ligand-binding sites. The TcdB-Fzd interactions inhibit canonical pathway activity, establishing a model of bacterial pathogen hijacking of Wnt pathway receptors through the evolution of effectors that cross-react with conserved domains of the Fzd family of proteins (60). Pathway redundancy conferred by conserved receptor domains is relevant to *E. chaffeensis* Wnt pathway manipulation, potentially explaining how RNA silencing of individual Fzd homologues did not completely inhibit *E. chaffeensis* infection. The current model of *E. chaffeensis* invasion of host cells holds that ehrlichial invasin EtpE interacts with glycophosphatidylinositol-anchored protein DNase X to trigger actin-mediated internalization and dampen the host innate immune response (3–6). DNase X^−/−^ cells are not completely resistant to infection, supporting our hypothesis that TRP120 as an adhesin initiates Wnt-dependent signaling that either directly or indirectly facilitates infection through initiation of phagocytosis; autophagy modulation, which our lab has previously investigated; or immunosuppression, which has been investigated in relation to other intracellular pathogens (61–64). CD147, also known as extracellular matrix metalloprotease inducer (EMMPRIN), is recruited to the EtpE-DNase X complex upon *E. chaffeensis* binding and entry, and it is hypothesized this transmembrane protein is central in relaying the phagocytosis signal to intracellular effectors (4). Interestingly, CD147 is upregulated by activation of canonical Wnt signaling in breast cancer cells, and silencing of CD147 has been shown to suppress β-catenin signaling (65, 66). This suggests CD147 may serve as a link between Wnt signaling activation by *E. chaffeensis* during early infection and the DNase X-dependent mechanism of bacterial invasion, but the relationship has not been investigated. There are likely multiple pathways activated that result in attachment and entry of *E. chaffeensis* It is also possible innate Wnt ligand-Fzd receptor signaling can modulate infection, which may explain why infection is significantly reduced when Fzd1, 2, 3, and 8 are silenced even though *E. chaffeensis* does not appear to strongly colocalize with those receptors during entry into the host cell.

*E. chaffeensis* was found to very strongly colocalize with the Wnt pathway coreceptor LRP5, and infection was significantly reduced in LRP5 and LRP6 RNA-silenced cells. These results are consistent with and expand upon our previous observation that silencing of LRP6 significantly reduces infection (22). The LRP coreceptors are typically attributed to the canonical Wnt signaling pathway and are activated by ligand-induced dimerization of the Fzd which triggers phosphorylation of LRP by kinases such as glycogen synthase kinases, G-protein-coupled receptor kinases, and casein kinases (67). Dvl is recruited to the phosphorylated receptor complex, and the intracellular signal cascade is initiated to free β-catenin from degradation. However, some reports have found LRP signaling to transduce through DAAM1 and RhoA, a Wnt/PCP pathway that controls the actin cytoskeleton (68). We have previously established that both canonical and noncanonical signaling pathways are necessary for full *E. chaffeensis* infection in THP-1 cells (22). LRP5 as a coreceptor for *E. chaffeensis* may explain how the bacterium both hijacks the signaling pathway during entry to attach to the host cell and primes it for infection by activating Wnt pathways. *E. chaffeensis* did not colocalize with LRP6 at early stages of infection, but silencing of LRP6 strongly reduced infection level in monocytes. While these data suggest LRP6 may not be involved as a receptor for *E. chaffeensis*, silencing of LRP6 may still significantly inhibit infection through sufficiently blocking canonical signaling in THP-1 cells, which could result in low infection levels in these cells. The respective role of either of these Wnt pathway coreceptors has not been investigated in the context of monocyte infections. Investigating the role of Wnt pathway coreceptors in infection will further elucidate the mechanism underlying noncanonical and canonical Wnt pathway manipulation by *E. chaffeensis*.

Colocalization of *E. chaffeensis* with Fzd2, 4, 5, 7, and 9 suggested these proteins serve as receptors for infection. Therefore, we investigated direct interaction of recombinant TRP120, for which a host receptor has not been identified, and the ECD of all Fzd homologues compared to interaction of Wnt5a with the homologues. Strong interactions identified by this screen include Fzd2, 4, 5, 7, and 9. Fzd5 and Fzd9 have repeatedly been identified by our group to be involved in *E. chaffeensis* infection, but the specific ehrlichial protein that may be interacting with the receptors has not been identified until now (22). Fzd10 appeared to weakly interact with rTRP120, but *E. chaffeensis* did not colocalize with the protein during infection, suggesting the direct binding is an artifact of the *in vitro* assay or that certain ligand-binding events may be at play during infection that prevent colocalization of *E. chaffeensis* and Fzd10. Altogether, identification of multiple Fzd homologues that interact with TRP120 suggests the bacterial protein is able to bind the conserved CRD of multiple receptors.

We chose to further investigate the model of TRP120-Fzd interaction by specifically studying Fzd5-TRP120 binding, as this Fzd has been shown to be involved in both canonical and noncanonical signaling and is involved in Wnt signaling control of the cytoskeleton (69). Fzd5 was also shown to regulate internalization of Escherichia coli by RAW264.7 macrophages (26). Treatment of cells with its ligand Wnt5a caused an increase in bacterial uptake by the cells and a decrease in killing of internalized bacteria. RNA-mediated silencing of Fzd5 also reduced the number of bacteria taken up by the macrophages. While a mechanism has yet to be established, it is likely that a Wnt5a-Fzd5 signaling axis regulates bacterial phagocytosis and can be manipulated by pathogens for entry and survival within a host cell (26). We investigated the colocalization of TRP120 and Fzd5 in our THP-1 model using rTRP120-FL-coated microspheres. TRP120 is expressed only by the dense-cored ehrlichiae, which means it is present on the surface of the bacterium and secreted into the host cell following internalization of the bacteria. However, our previous studies have indicated that TRP120 is difficult to visualize by fluorescence microscopy prior to approximately 24 hpi. The protein-coated microspheres serve as a proxy for infection and can be more easily visualized to investigate Fzd and TRP120 colocalization. Indeed, we show that TRP120-coated microspheres can colocalize with Fzd5, indicating that the microspheres are either adhered to or internalized by the THP-1 cells. Additionally, Fzd5 coimmunoprecipitates with TRP120 from infected cell lysate, which demonstrates the interaction is happening during *E. chaffeensis* infection. We further investigated this interaction by determining the binding affinity of TRP120-Fzd5. The full-length recombinant protein interacts with the Fzd5-CRD with a binding affinity of approximately 46.3 μM, and the TRD binds with an affinity of approximately 1.35 μM. The relative 10-fold increase in binding affinity of the full-length protein relative to the 2xTR domain construct indicates that either a tertiary structure or amino acids outside the TRD are necessary for enhanced binding affinity. Multiple studies using various methods have investigated the affinity of Wnt ligands for Fzd receptors, and the results indicate affinity within the range of 1 to 10 nM (70). This stronger affinity of Wnt ligands for Fzd receptors relative to the affinity of TRP120 and Fzd brings to question whether competition between ligand and ligand mimic for binding of the receptor may occur and whether innate ligand-receptor interaction could still facilitate internalization of *E. chaffeensis* because of the resultant pathway activity that is initiated.

TRP120 is an intrinsically disordered protein and as such has been shown to possess eukaryotic SLiMs that enable a multitude of interactions with host cells. We identified significant amino acid sequence similarity between the receptor-binding site 1 of Wnt5a/Wnt5b and TRP120. Wnt5a is primarily studied in the context of noncanonical signaling as a driver of cell motility pathways but can also activate canonical signaling (45, 71–73). This duality can be explained in part by the observation that all Fzd homologues interact with Wnt5a in cell-type-specific contexts (43–51). Mimicry of a dual-function Wnt ligand may shed light on the mechanism of regulation of multiple Wnt signaling pathways by TRP120. To supplement the sequence homology identified by BLAST, we utilized the ISM to predict functional similarity between TRP120 and XWnt8, the same ligand that was used in structure studies of Wnt/Fzd interaction (52). ISM predicts shared biological activity between two macromolecules (e.g., proteins or DNA) as dictated by each molecule’s respective electromagnetic energy (74). It was recently used to identify common information shared by Zika virus proteins and host proteins that resulted in autoantibody production during viral infection (75). The common information between TRP120 and XWnt8 was related to the receptor-binding site 1 within XWnt8, suggesting the shared biological activity is binding of a common receptor which we hypothesize to be the Fzd-ECD.

We previously established that *E. chaffeensis* can activate Wnt signaling by inducing nuclear translocation of the canonical Wnt pathway transcription factor β-catenin (22). Therefore, this positive control sufficed for our studies of Wnt pathway activation. We demonstrated that rTRP12-FL can induce β-catenin activation to a similar degree as *E. chaffeensis* within 3 h of treatment. This time frame was chosen as it is the earliest that β-catenin nuclear translocation is detected during *E. chaffeensis* infection, and it mimics the period of time that *E. chaffeensis* can remain extracellular and viable (22, 40). Based on our homology data demonstrating a Wnt SLiM in the TRD of TRP120 that resembles receptor-binding site 1 of Wnt ligands, we used a synthetic 21-mer peptide to demonstrate the TRP120 Wnt SLiM alone can stimulate Wnt pathway activity. We chose to mutate the seven amino acids within the Wnt SLiM peptide that are homologous to receptor-binding site 1 in the negative-control Wnt SLiM peptide mutant to a series of alanine and glycine residues, as this was deemed a desirable mutation for synthesis and solubilization of the peptide. The receptor-binding site 1 of Wnt ligands is the site of a lipidated serine (52). This palmitoleic acid has been shown to be necessary for secretion of Wnt ligands, rendering them inactive if lipidation does not occur (76, 77). Additionally, this lipid directly engages a groove in the receptor which has established it as a critical posttranslational modification for receptor binding and activation of signaling (52). Not only did we show that solubilized rTRP120-FL could activate canonical Wnt signaling, we also demonstrated that a Wnt SLiM peptide is sufficient for induction of β-catenin nuclear localization, which suggests that Wnt ligand mimics, let alone Wnt ligands, may be functional without lipidation. The idea of activating canonical Wnt signaling by exploiting receptor binding is not novel; for example, synthetic decapeptides resembling Wnt signaling inhibitor DKK1 have been shown to stimulate Wnt pathway activity through manipulating the DKK1-LRP6 binding interface (78). However, the notion of linear motifs derived from conserved portions of Wnt ligands that can activate β-catenin nuclear translocation in the absence of other modifications or signals has not been reported in eukaryotic models. Furthermore, this is the first report of eukaryotic Wnt SLiM mimicry in bacterial proteomes, a novel virulence strategy by obligate intracellular pathogens. The intrinsically disordered TRP120 is a unique tool in investigation of the underlying molecular mechanism for such virulence strategies.

To demonstrate that disruption of the Wnt SLiM in full-length TRP120 results in an inability to activate signaling, we made several attempts to create a recombinant protein with a mutated Wnt SLiM but were unsuccessful, as manipulation of amino acids in the TRD of TRP120 changed the biochemical properties of the protein and made expression and purification difficult. To circumvent this, we used an antibody that would recognize the Wnt SLiM and inactivate the sequence by blockage. Indeed, we showed antibody binding and blocking of the Wnt SLiM inhibits both rTRP120-FL and *E. chaffeensis* activation of Wnt signaling. Furthermore, inhibition of this region during infection significantly reduces the infection level 24 hpi, indicating an impaired ability of *E. chaffeensis* to establish infection in the host cell. This not only indicates that the TRP120 Wnt SLiM is necessary for full *E. chaffeensis* infection but also suggests that it is a potential therapeutic target and further demonstrates that the Wnt pathway is used by this pathogen for survival within the host cell. Other obligate intracellular pathogens including *Chlamydia* and *Rickettsia* spp. also demonstrate activation of Wnt signaling in their respective host cells, and inhibition of signaling impairs bacterial growth (63, 79, 80). Further studies to address the role of Wnt signaling during the life cycle of *E. chaffeensis* and various obligate intracellular bacteria, as well as viral pathogens, could identify novel antimicrobial therapeutics targets.

Here, we established a model of eukaryotic protein mimicry for manipulation of the eukaryotic signaling pathway in the host cell of *E. chaffeensis* during infection. *E. chaffeensis* has proven to manipulate the host cell at a number of subcellular levels. This study identifies a receptor for the previously observed adhesin activity of TRP120 and identifies a mechanism of Wnt signaling pathway during infection. Further studies to investigate the link between Wnt signaling and bacterial phagocytosis will identify mechanisms of pathogenesis and highlight potential targets for novel therapeutics.

## MATERIALS AND METHODS

### Cell culture and cultivation of Ehrlichia chaffeensis.

Human monocytic leukemia cell line THP-1 (ATCC TIB-202) was cultured and propagated per ATCC recommendations. Cultivation of Ehrlichia chaffeensis strain Arkansas in THP-1 cells was performed as previously described (81).

### Antibodies, primers, and synthetic peptides.

Primary antibodies targeting eukaryotic proteins used in this study for immunofluorescence microscopy were mouse α-Fzd1 (catalog no. sc-398082), mouse α-Fzd3 (sc-376105), and mouse α-Fzd7 (sc-293261; Santa Cruz Biotechnology, Dallas, TX); rabbit α-Fzd2 (catalog no. NBP2-33842), rabbit α-Fzd8 (catalog no. NBP2-75493), and rabbit α-Fzd10 (NBP2-23659; Novus Biologicals, Centennial, CO); rabbit α-Fzd4 (catalog no. A8161; Abclonal, Woburn, MA); rabbit α-Fzd6 (catalog no. PA1-32780; Invitrogen, Waltham, MA); rabbit α-Fzd9 (catalog no. AV-41253; Sigma); rabbit α-Fzd5 (catalog no. 5266S), rabbit α-LRP5 (5440S), rabbit α-LRP6 (3395S), and mouse α-β-catenin (2677S; Cell Signaling Technology, Danvers MA); and rabbit α-β-catenin (catalog no. 600-401-C68; Rockland Immunochemicals, Limerick, PA). Mouse α-His (catalog no. A00612; GenScript, Piscataway, NJ) was used for immunofluorescent labeling of purified recombinant proteins. Primary antibodies targeting eukaryotic proteins used in this study for ELISA were mouse α-Wnt5a (catalog no. MA515502; Thermo Fisher Scientific, Waltham, MA). Primary antibodies targeting eukaryotic proteins used in this study for Western blotting were rabbit α-Fzd1 (catalog no. AB126262; Abcam, Cambridge, MA); rat α-Fzd2 (catalog no. sc-74019), mouse α-Fzd3 (sc-376105), mouse α-Fzd6 (sc-393791), mouse α-Fzd7 (sc-293261), mouse α-ROR1 (sc-130386), mouse α-Dvl1 (sc-8025), and mouse α-Dvl2 (sc-39030; Santa Cruz); mouse α-Fzd4 (catalog no. H00008322-M02A; Abnova, Taipei, Taiwan); rabbit α-Fzd8 (catalog no. NBP2-75493) and rabbit α-Fzd10 (NBP223659; Novus); rabbit α-Fzd9 (catalog no. AB108628; Abcam); rabbit α-Fzd5, rabbit α-LRP5, rabbit α-LRP6, and rabbit α-ROR2 (catalog no. 88639S; Cell Signaling); and rabbit anti-glyceraldehyde-3-phosphate dehydrogenase (α-GAPDH; catalog no. 10494-1-AP; Proteintech, Rosemont, IL). Anti-*E. chaffeensis* antibodies used in this study include human monoclonal antibody 15 (HuMAb 15) targeting *E. chaffeensis* outer membrane protein 19 (54), rabbit α-DSB (82), and rabbit α-TRP120-I1 (55). Rabbit α-TRP120-Wnt-SLiM antiserum was commercially generated against a synthetic peptide resembling TRP120 amino acids 84 to 104 (Biomatik, Cambridge, ON, Canada). Synthetic peptides used in this study were commercially generated (Biomatik) and are DLQDVASHESGVSDQPAQVVT (wild type) and DLQDVASGAGAGAGQPAQVVT (mutant).

### Synchronous *E. chaffeensis* infection.

Cell-free *E. chaffeensis* was used to establish synchronized infection in THP-1 cells. THP-1 cells 100% infected with *E. chaffeensis* as determined by modified Wright-Giemsa staining were harvested by centrifuging, resuspended in ice-cold phosphate-buffered saline (PBS), and lysed by homogenization with the BeadBug Microtube homogenizer (Benchmark, Edison, NJ) (speed 3,000 rpm for 10 s, twice). Cellular lysate was separated from *E. chaffeensis* by ice-cold centrifugation at 100 × *g* followed by 200 × *g* for 10 min each, followed by pelleting of cell-free *E. chaffeensis* by ice-cold centrifugation at 10,000 × *g*. The bacterial pellet was resuspended in ice-cold PBS before being added to THP-1 cells at a multiplicity of infection (MOI) of 100 to 200. To derive MOI of cell-free *E. chaffeensis* from initial population of 100% infected THP-1 cells, the following calculation was used: *X* = MOI(*Z*)/41.4, where *X* is the number of infected cells from which cell-free *E. chaffeensis* should be made for desired MOI in *Z* number of target cells (83). To control for contaminating host cell lysate in the cell-free *E. chaffeensis*, uninfected THP-1 cells were processed identically to the infected cells and the resultant lysate was used for mock infections.

### Immunofluorescence microscopy.

THP-1 cells to be analyzed by immunofluorescence microscopy were washed once with room-temperature PBS, and 50 μl/sample was cytocentrifuged onto glass slides. Cells were fixed with 4% paraformaldehyde for 15 min, blocked and permeabilized in 0.1% Triton X-100 diluted in blocking buffer (2% bovine serum albumin [BSA] in PBS) for 30 min, stained with respective primary antibodies diluted 1:25 to 1:50 in blocking buffer (2% BSA in PBS) for 1 h, incubated with Alexa Fluor-conjugated secondary antibodies goat α-rabbit, goat α-mouse, or goat α-human (Thermo Fisher) diluted 1:200 in blocking buffer for 30 min, and mounted with ProLong Gold Antifade with DAPI (4′,6-diamidino-2-phenylindole; Thermo Fisher). Slides were imaged on a Zeiss LSM 880 confocal laser scanning microscope. Three experimental replicates were done for each immunofluorescence experiment. MOC quantification and line intensity profiles were generated using representative images using FIJI (FIJI is Just Image J) (84). Mean fluorescence intensity per area (MFI/area) quantifications were performed in FIJI by designating all pixels of the 405-nm channel (DAPI, nucleus) image as regions of interest (ROIs) and measuring the MFI of the pixels within that ROI in the corresponding 488-nm channel (green, β-catenin) image. MFI/area was calculated by dividing the MFI of the 488-nm channel by the area of the region of interest measured to normalize for variations in cell number and nucleus size across samples and groups. Five to seven fields containing at least 10 infected or treated cells were captured per replicate and used for quantification.

### Recombinant protein expression, purification, and quantification.

Escherichia coli TOP10 transformed with either pBAD/Thio-TOPO expression vector containing the full-length TRP120 gene upstream of a 6×His tag (55) or empty vector was inoculated into Terrific Broth (TB) (Corning Inc., Corning, NY), grown overnight, and diluted 1:50 in fresh TB. Cultures were induced with 20% arabinose for 3 h and harvested by centrifuging at 4,000 × *g* for 20 min at 4°C. The pellets were suspended in ice-cold lysis buffer (50 mM Tris, pH 7.5, 150 mM NaCl, 2 mM dithiothreitol [DTT], 2 mM MgCl_2_, 5 mM EDTA, 5 mM imidazole) and homogenized using a BeadBeater (BioSpec, Bartlesville, OK). Lysate was collected by ice-cold centrifugation at 12,000 × *g*, run over 1 ml of cOmplete His-tag purification resin (Roche, Basel, Switzerland) packed in a Talon disposable gravity column prepared per manufacturer’s protocol (TaKaRa Bio, Mountain View, CA), and washed with a series of increasingly concentrated imidazole solution washes (5 mM to 40 mM imidazole; 50 mM Tris, pH 7.5, 150 mM NaCl, 2 mM DTT, 2 mM MgCl_2_, 5 mM EDTA). Recombinant protein was sequentially eluted with a 500 mM imidazole solution. Eluate samples were analyzed for purity by SDS-PAGE followed by AcquaStain protein gel stain (BulldogBio, Portsmouth, NH), and then eluates were dialyzed for 24 h in ice-cold PBS and quantified by bicinchoninic acid assay (Thermo Fisher).

### Indirect enzyme-linked immunosorbent assay (ELISA).

Nunc MaxiSorp 96-well plates (Thermo Fisher) were coated with 100 ng of commercially available recombinant Frizzled cysteine-rich domain-truncated proteins (Fzd1, Fzd2, Fzd4, Fzd5, Fzd7, Fzd8, Fzd9, Fzd10, R&D Systems, Minneapolis, MN; Fzd3, Fzd6, Abcam) diluted in 100 μl PBS by overnight incubation at 4°C. Wells were blocked with 100 μl of 5% horse serum in 5% Tris-buffered saline (TBST) for 1 h at room temperature with gentle shaking (300 rpm). Recombinant TRP120 or rWnt5a (R&D Systems) (500 ng) diluted in 100 μl PBS was added to each rFzd well and incubated for 1 h at room temperature with gentle shaking. Primary antibodies diluted (1:5,000) in blocking buffer were added to each well and incubated for 1 h at room temperature, and then a secondary alkaline phosphatase-conjugated antibody (1:10,000; KPL, Gaithersburg, MD) was added and incubated for 1 h, both with gentle shaking. Wells were washed 2 to 3 times with 200 μl 0.2% TBST in between each incubation. Wells were developed with 50 μl BluePhos alkaline phosphatase substrate (KPL) at room temperature in the dark with gentle shaking, and color development was measured at *A*_650_ using a VersaMax microplate reader (Molecular Devices, Sunnyvale, CA). Data were recorded and analyzed by SoftmaxPro v. 7.0 (Molecular Devices). *A*_650_ readings represent blank-subtracted values of respective wells.

### Protein-coated microsphere assay.

One-micrometer sulfate microspheres (Invitrogen) were diluted in PBS to a final multiplicity of treatment of 50 microspheres per cell, or 5 × 10^6^ microspheres per 10^5^ THP-1 cells per group. After dilution, microspheres were briefly sonicated, and purified rTRP120 or negative-control rTrx was added at 2 μg per 5 × 10^6^ microspheres to a total volume of 25 μl. Microspheres were incubated end-over-end at room temperature with recombinant protein for adsorption to occur. Microspheres were collected by centrifugation at 12,000 × *g* for 15 min, washed once with 0.1× PBS, and resuspended in equal-volume 0.1× PBS plus 1% BSA plus 0.01% Tween 20. Sufficient protein coating of microspheres was evaluated by dot blotting of microsphere sample after protein adsorption. Microspheres were briefly sonicated just before treatment and added to THP-1 cells that were plated 1 day prior. Cells were shaken at 300 rpm for 1 min and incubated for 1 h at 37°C. Cells were washed once with PBS and then processed for analysis by immunofluorescence microscopy.

### Protein coimmunoprecipitation.

Coimmunoprecipitation of TRP120 and host proteins was performed using the Pierce coimmunoprecipitation kit (Thermo Fisher) per manufacturer’s instructions. Lysate was harvested from THP-1 cells 70 h post-synchronous *E. chaffeensis* infection (MOI 50). TRP120 was immunoprecipitated with rabbit α-TRP120-I1. Twenty-five microliters of immunoprecipitated eluates and 30 μg of starting input were processed for Western blotting as described below.

### Surface plasmon resonance.

Binding affinity of rTRP120 and rFZD-ECD truncate proteins was determined by surface plasmon resonance using a Biacore T100 (Cytiva, Marlborough, MA). Ligands (purified His-tagged rTRP120-FL and His-tagged rTRP120-TRD) and analytes (rFZD5-ECD and rFZD8-ECD [R&D Systems]) were dialyzed in running buffer (100 mM NaPO_4_, pH 7.4, 400 mM NaCl, 40 μM EDTA, 0.005% Tween 20). Each running cycle began with a 500 μM NiCl_2_ chip charging. Ligands (0.1 μM) were immobilized on flow cell 2 of Biacore sensor chip NTA with a flow rate of 30 μl/min for 100 s. A range of concentrations of respective analytes (0 to 1,600 nM) were injected at a flow rate of 30 μl/min with contact time of 360 s and a dissociation time of 300 s. The chip was regenerated with 350 mM EDTA. Curve fitting was performed using a 1:1 surface-bound curve fitting model, and the binding constant (*K_D_*) was reported to be uniquely determined as a quality control measure.

### Wnt target gene expression.

The human Wnt signaling target PCR array profiles the expression of 84 key genes responsive to Wnt signal transduction, including Wnt signaling pathway transcription factors and highly relevant target genes to analyze Wnt pathway status. PCR arrays were performed according to the PCR array handbook from the manufacturer (Qiagen). In brief, uninfected and *E. chaffeensis*-infected and *E. chaffeensis* Wnt SLiM peptide-stimulated THP-1 cells were harvested 12 h postinfection. Total RNA purification was performed using the RNeasy minikit (Qiagen) as previously described. Minor modifications to the protocol included centrifugations for 30 s at 12,000 rpm, 20 to 24°C; additional RW1 wash was added prior to RPE washes; and an additional 500 μl RPE wash was added prior to drying the membrane. Prior to elution, the spin column was centrifuged at 14,500 rpm for 1 min to dry the membrane. During RNA purification, on-column DNA digestion was performed using the RNase-free DNase set (Qiagen). The concentration and purity were determined by measuring the absorbance using a NanoDrop 100 spectrophotometer (Thermo Scientific, West Palm Beach, FL), and rRNA band integrity was verified by running an aliquot of each RNA sample on an RNA FlashGel (Lonza, Rockland, ME). Genomic DNA was eliminated, and cDNA was synthesized from 0.5 μg of total RNA using the RT^2^ first-strand kit (Qiagen). Real-time PCR was performed using RT^2^ Profiler PCR array in combination with RT^2^ SYBR green master mix (Qiagen) on a QuantStudio 6 Flex real-time PCR system. Cycling conditions were as follows: 95°C for 10 min and 40 cycles of 95°C for 30 s, 60°C for 1 min, 72°C for 30 s, and 65°C for 30 s. The ramp rate was set to 1°C/s. The real-time cycler software QuantStudio Real-Time PCR v1.3 was used for PCR and data collection. The baseline was set automatically, the threshold was defined manually, and then the threshold cycle (*C_T_*) for each well was calculated by RealPlex. The threshold was set in the proper location and at the same level for all PCR arrays in the same analysis so that the values of the positive PCR control (PPC) assays on all arrays were between 18 *C_T_* and 22 *C_T_*. The *C_T_* values for all wells were exported for analysis using web-based PCR array data analysis software (version 3.5; SABiosciences). PCR array quality checks were performed by the software before data analysis, including PCR array reproducibility, reverse transcription efficiency control (RTC), human genomic DNA contamination control (HGDC), and PPC.

### siRNA gene knockdown and *E. chaffeensis* enumeration by quantitative PCR.

Gene silencing by small interfering RNA followed by qPCR of the ehrlichial *dsb* gene was performed as previously described (85). All siRNAs are ON-TARGETplus SMARTpool siRNA (Dharmacon, Lafayette, CO) and are described in Table S1 in the supplemental material.

10.1128/mSphere.00216-21.2 TABLE S1 Sequences of siRNA used in this study. Download Table S1, DOCX file, 0.02 MB. Copyright © 2021 Rogan et al. 2021 Rogan et al. https://creativecommons.org/licenses/by/4.0/ This content is distributed under the terms of the Creative Commons Attribution 4.0 International license.

### Western blotting.

THP-1 cell lysates were prepared as previously described (86). Cell lysates (30 μg) were separated by SDS-PAGE and transferred to a nitrocellulose membrane that was blocked for 1 h in blocking buffer (5% milk plus 0.1% Tween 20 in Tris-buffered saline [TBST]), incubated with primary antibodies (diluted per manufacturer’s recommendation in blocking buffer) overnight, and immunoblotted with horseradish peroxidase-conjugated secondary antibodies goat α-rabbit, goat α-mouse, or goat α-rat (SeraCare, Milford, MA) (diluted 1:10,000 in blocking buffer) for 1 h. Membranes were washed three times for 15 min each in 0.1% TBST between incubations. Western blots were developed following the addition of SuperSignal West Dura chemiluminescent substrate (Thermo Fisher) and visualized with ChemiDoc-It^2^ imager (UVP, Upland, CA). Immunoblots were stripped with Restore Western blot stripping buffer (Thermo Fisher) and reprobed with control housekeeping primary antibodies. For β-catenin Western blots, THP-1 cell lysates were prepared using CytoBuster protein extraction reagent (Novagen/EMD, Gibbstown, NJ), separated by sodium dodecyl sulfate-polyacrylamide gel electrophoresis (SDS-PAGE), and transferred to a nitrocellulose membrane. Western immunoblotting was performed with mouse anti-human β-catenin and GAPDH (Pierce, Rockford, IL) and rabbit anti-human phospho-β-catenin and nonphospho-β-catenin (Cell Signaling, Beverly, MA), and blots were developed and imaged as described above. Image acquisition and densitometry analysis were performed using VisionWorks software (UVP).

### Protein sequence homology identification.

TRP120 protein sequence (NCBI gene accession number AAO12927.1) and each of the Homo sapiens Wnt proteins were submitted to NCBI Protein Basic Local Alignment Search Tool (Protein BLAST) for alignment of two sequences. Results returned indicating significant homology between TRP120 sequence and Wnt sequence were down-sampled for results in which the region of the Wnt protein sequence displaying homology with the TRP120 protein sequence comprised at least one of the two receptor-binding sites (52).

### Informational spectrum method analysis.

The *in silico* analysis was performed by the Biomed Protection (biomedprotection.com) proprietary wEB platform. This bioinformatics platform is based on the informational spectrum method (ISM) representing a virtual spectroscopy method for analysis of protein-protein interactions. According to the ISM technique that has been described in detail previously (53), protein sequences are transformed into signals by assignment of numerical values of each amino acid. These values correspond to the electron-ion interaction potential determining electronic properties of amino acids (87, 88). The signal obtained is then decomposed in periodical function by Fourier transformation. The result is a series of frequencies and their amplitudes. The obtained frequencies correspond to the distribution of structural motifs with defined electronic characteristics that are responsible for biological function of the sequence. When comparing interacting proteins, the ISM technique allows detection of code/frequency pairs which are specific for their interaction (53).

### β-Catenin nuclear translocation assay.

Low-passage-number (between 5 and 9) THP-1 cells were grown to a density of 500,000 cells/ml and treated with 2 μg/ml rTRP120, rTrx, or synthetic peptides or infected with cell-free *E. chaffeensis* for 3 h at 37°C. Fifty-microliter samples were processed for immunofluorescence microscopy analysis and quantification.

### Antibody inhibition of rTRP120 or *E. chaffeensis* Wnt pathway activation.

Antibodies or preimmune sera were diluted 1:200 in THP-1 culture medium. Low-passage-number THP-1 cells were plated on a 96-well round-bottom plate at a density of 100,000 cells in 50 μl of each antibody dilution and incubated for 2.5 h at 37°C. Two micrograms/milliliter rTRP120 or cell-free *E. chaffeensis* (MOI 100) diluted in culture medium was added to antibody-treated cells and incubated for another 3 h. Cells were washed once with PBS and processed for immunofluorescence microscopy or enumeration of *E. chaffeensis* by qPCR of the *dsb* gene.

### Rickettsia parkeri infection of THP-1 cells.

Two milliliters of low-passage-number THP-1 cells was plated at a density of 10^6^ cells/ml per well on a 6-well plate. From a Rickettsia parkeri strain Atlantic Rainforest-like stock vial, 250 μl was diluted in 25 ml culture medium. Each well was then inoculated with 1 ml of the *R. parkeri* dilution. Once sealed with Parafilm, plates were centrifuged at 2,000 rpm for 5 min to facilitate attachment of bacteria to THP-1 cells. Cells were then harvested and processed for immunofluorescence microscopy staining as described above. *R. parkeri* was tagged with serum from guinea pigs infected with R. rickettsii (1:16,000 titer), and Alexa Fluor 488 goat α-guinea pig IgG (Invitrogen; catalog no A11073) at 1:1,000 titer was used as the secondary antibody.
