# Supplementary figures and images for: Ehrlichia chaffeensis TRP120 Is a Wnt Ligand Mimetic That Interacts with Wnt Receptors and Contains a Novel Repetitive Short Linear Motif That Activates Wnt Signaling
Source: mSphere. 2021 Apr 21;6(2):e00216-21. doi: 10.1128/mSphere.00216-21 (PMC8546699; doi:10.1128/mSphere.00216-21)

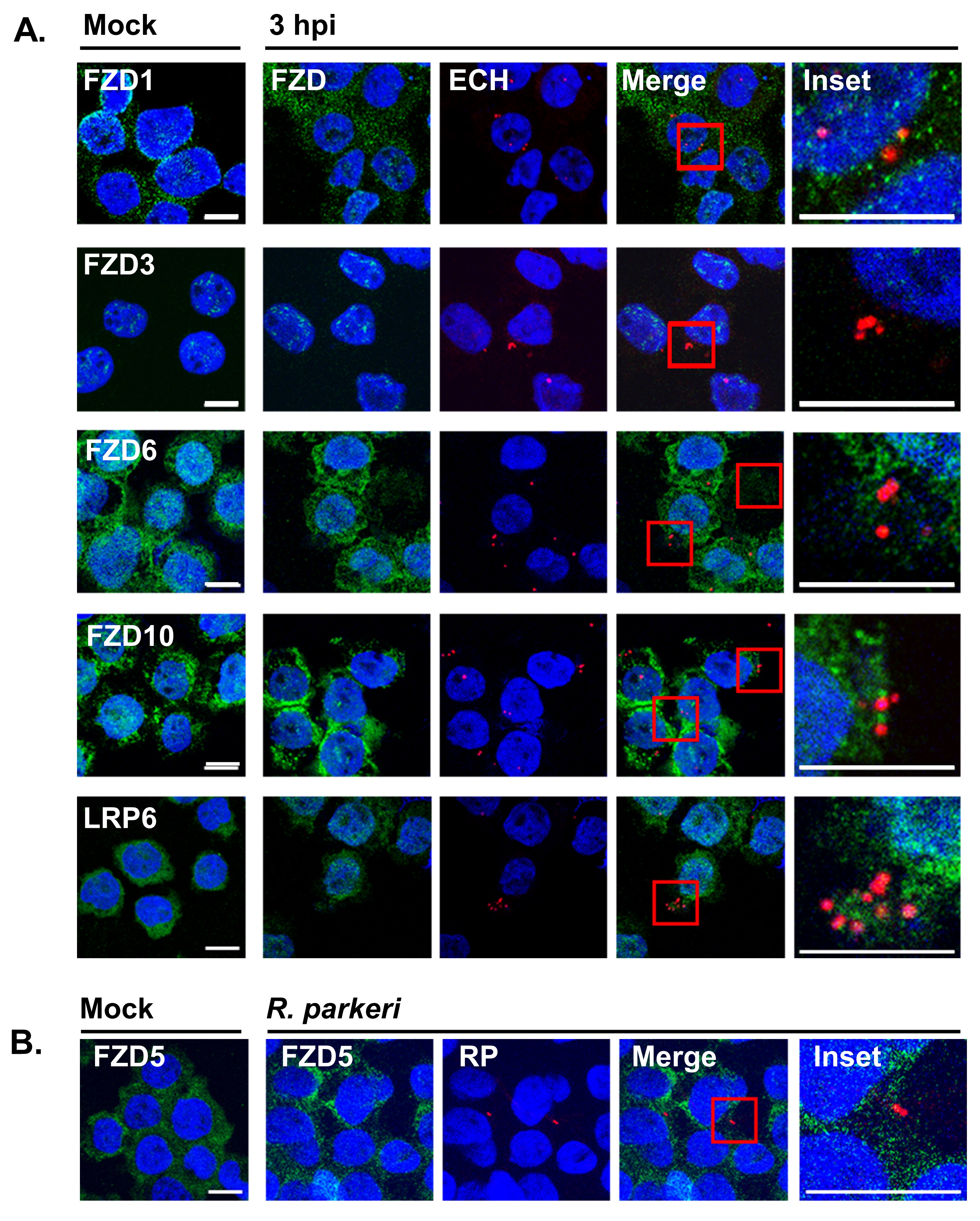

Supplement: FIG S1 [file msphere.00216-21-sf001.jpg]
