# Supplementary material for: Ehrlichia chaffeensis TRP120 Is a Wnt Ligand Mimetic That Interacts with Wnt Receptors and Contains a Novel Repetitive Short Linear Motif That Activates Wnt Signaling
Source: mSphere. 2021 Apr 21;6(2):e00216-21. doi: 10.1128/mSphere.00216-21 (PMC8546699; doi:10.1128/mSphere.00216-21)
Supplement: TABLE S2 [file msphere.00216-21-st002.docx]

| **Gene Symbol** | ***E.ch.* - 12h** | **E.ch. Wnt peptide - 12h** |
| --- | --- | --- |
| ABCB1 | **10.33** | **14.14** |
| AHR | **14.46** | **19.22** |
| ANGPTL4 | **-16.42** | **-16.81** |
| ANTXR1 | **-15.81** | **-19.13** |
| AXIN2 | **6.07** | **6.46** |
| BGLAP | **4.84** | **3.76** |
| BIRC5 | **7.76** | **15.75** |
| BMP4 | **6.54** | **14.52** |
| BTRC | **24.02** | **19.30** |
| CACNA2D3 | **25.17** | **19.73** |
| CCND1 | **-29.91** | **-16.14** |
| CCND2 | **-29.28** | **-15.22** |
| CD44 | **13.46** | **16.77** |
| CDH1 | **16.02** | **17.79** |
| CDKN2A | **-18.11** | **-19.79** |
| CDON | **-21.49** | **-20.56** |
| CEBPD | **4.11** | **3.53** |
| CTGF | **4.50** | **4.47** |
| CUBN | **6.50** | **13.69** |
| DAB2 | **6.57** | **14.59** |
| DKK1 | **18.97** | **16.23** |
| DLK1 | **54.81** | **45.24** |
| DPP10 | **-24.06** | **-15.91** |
| EFNB1 | **-30.22** | **-13.96** |
| EGFR | **-5314.06** | **-19.84** |
| EGR1 | **-2257.64** | **-19.54** |
| ETS2 | **15.03** | 1.39 |
| FGF20 | **17.36** | 1.37 |
| FGF4 | **13.36** | **219.40** |
| FGF7 | **6.19** | **228.71** |
| FGF9 | **-21.55** | **34.23** |
| FN1 | **-23.02** | **31.44** |
| FOSL1 | **-1047.40** | **-23.27** |
| FST | **-513.99** | **-21.46** |
| FZD7 | 1.07 | **-4.36** |
| GDF5 | -1.48 | **-5.11** |
| GDNF | **-2096.26** | **-12.83** |
| GJA1 | **-2086.11** | **-13.18** |
| ID2 | **20.29** | 1.89 |
| IGF1 | **15.38** | 1.50 |
| IGF2 | **6.47** | **219.40** |
| IL6 | **5.68** | **228.71** |
| IRS1 | **-13.39** | **37.67** |
| JAG1 | **-15.37** | **51.39** |
| KLF5 | **-761.45** | **-19.98** |
| LEF1 | **-618.49** | **-10.91** |
| LRP1 | 1.07 | **-3.54** |
| MET | 1.22 | **-3.27** |
| MMP2 | -1.90 | **-2.09** |
| MMP7 | **-3.06** | -1.94 |
| MMP9 | **-62.93** | **-49.34** |
| MYC | **-48.22** | **-68.34** |
| NANOG | **-2.62** | **-3.71** |
| NRCAM | **-2.58** | **-5.27** |
| NRP1 | **4.20** | **5.14** |
| NTRK2 | **7.79** | **7.21** |
| PDGFRA | **4.61** | **4.51** |
| PITX2 | **4.54** | **4.77** |
| PLAUR | **-3.63** | **-2.48** |
| POU5F1 | **-2.23** | **-3.02** |
| PPAP2B | **-4.49** | **-3.66** |
| PPARD | -1.08 | -1.86 |
| PTCH1 | **-44.90** | **-53.88** |
| PTGS2 | **-59.16** | **-70.31** |
| RUNX2 | **-2.92** | **-3.55** |
| SFRP2 | **-2.85** | **-2.96** |
| SIX1 | **4.08** | **4.31** |
| SMO | **8.42** | **7.34** |
| SOX2 | **6.01** | **5.57** |
| SOX9 | **7.68** | **6.47** |
| T | **-3.49** | **-3.27** |
| TCF4 | -1.97 | -1.50 |
| TCF7 | **4.73** | **3.55** |
| TCF7L1 | **3.63** | **4.64** |
| TCF7L2 | 1.87 | 1.72 |
| TGFB3 | **-513.99** | 1.71 |
| TLE1 | **-153.23** | **-95.31** |
| TWIST1 | **-143.97** | **-159.41** |
| VEGFA | **3.32** | **3.05** |
| WISP1 | **3.10** | **2.58** |
| WISP2 | **18.56** | **10.83** |
| WNT3A | **30.18** | **22.05** |
| WNT5A | 1.08 | 1.62 |
| WNT9A | **4.89** | **3.63** |
| ACTB | **4.98** | **4.89** |
| B2M | **7.01** | **8.78** |
| GAPDH | 1.55 | 1.43 |
| HPRT1 | **2.35** | 1.66 |
| RPLP0 | **-127.61** | **-101.87** |
