# Supplementary material for: Ehrlichia chaffeensis TRP120 Is a Wnt Ligand Mimetic That Interacts with Wnt Receptors and Contains a Novel Repetitive Short Linear Motif That Activates Wnt Signaling
Source: mSphere. 2021 Apr 21;6(2):e00216-21. doi: 10.1128/mSphere.00216-21 (PMC8546699; doi:10.1128/mSphere.00216-21)
Supplement: TABLE S1 [file msphere.00216-21-st001.docx]

| **Target** | **Gene ID** | **Sequence** |
| --- | --- | --- |
| Scramble |  | UGGUUUACAUGUCGACUAA |
|  |  | UGGUUUACAUGUUGUGUGA |
|  |  | UGGUUUACAUGUUUUCUGA |
|  |  | UGGUUUACAUGUUUUCCUA |
| FZD1 | 8321 | GUUCUACCCUCUAGUGAAA |
|  |  | UUACGUACCUGGUGGACAU |
|  |  | GAAGCCAACUCACAGUAUU |
|  |  | GCAAGACCCUCAACUCCUG |
| FZD2 | 2535 | CCACGUACUUGGUAGACAU |
|  |  | GAACUGCGCUUCUUCCUGU |
|  |  | GGAGGAAGUUCUACACUCG |
|  |  | GCUACAAGUUUCUGGGCGA |
| FZD3 | 7976 | CCAAAUACUCCUAUCAUAA |
|  |  | ACAGAUCACUCCAGGCAUA |
|  |  | GUUCGAAGCUCAUGGAGAU |
|  |  | UGAUUGAUGUCACAAGAUU |
| FZD4 | 8322 | GAUCGAUUCUUCUAGGUUU |
|  |  | UCACACCGCUCAUCCAGUA |
|  |  | GGACAAAGACAGACAAGUU |
|  |  | CCAAGGAGUUCACUGAUAU |
| FZD5 | 7855 | GCAUUGUGGUGGCCUGCUA |
|  |  | GCACAUGCCCAACCAGUUC |
|  |  | AAAUCACGGUGCCCAUGUG |
|  |  | GAUCCGCAUCGGCAUCUUC |
| FZD6 | 8323 | CCAGAGAGACCAAUUAUAU |
|  |  | UCGCAAAUCUGGAAUGUUC |
|  |  | GAAGGAAGGAUUAGUCCAA |
|  |  | CAGUGAAAGUCGAAGAGUA |
| FZD7 | 8324 | UCAAGUACCUGAUGACCAU |
|  |  | GUUCGUCUACCUCUUCAUA |
|  |  | UGAUGUACUUUAAGGAGGA |
|  |  | AGGCAUAACUGUGACGAAA |
| FZD8 | 8325 | AGACAGGCCAGAUCGCUAA |
|  |  | ACACCUACAUGCCCAAUCA |
|  |  | UCACCGUGCCGCUGUGUAA |
|  |  | CGGCGAGCUCCGUGUCUUA |
| FZD9 | 8326 | GCACGCACUGCCACUAUAA |
|  |  | CGGCAGCUAUUUCCACAUG |
|  |  | CUGCCGAGCUAGCGGAGUU |
|  |  | GCGAGAACCCCGAGAAGUU |
| FZD10 | 11211 | GCUACAACAUGACUCGUAU |
|  |  | GUAUCGGGCUCUUCUCUGU |
|  |  | UGUGAUCGCCUGCUACUUU |
|  |  | GGUGUGCAGCCGUAGGUUA |
| LRP5 | 4041 | CGUCAAAGCCAUCGACUAU |
|  |  | CGUCAUGGGUGGUGUCUAU |
|  |  | GGACGGACCUACGGAGGAU |
|  |  | GUACAGGCCCUACAUCAUU |
| LRP6 | 4040 | GCAGAUAUCAGACGAAUUU |
|  |  | CAGAUGAACUGGAUUGUUA |
|  |  | CCACAGAGCGAUCACAUUA |
|  |  | GCUCAACCGUGAAGUUAUA |
| ROR1 | 4919 | UGACUUGUGUCGCGAUGAA |
|  |  | GGCAGAUCCUAUAAAUAAA |
|  |  | CAUCAAUGGAUACCCAAUA |
|  |  | GGAGAGCAACUUCAUGUAA |
| ROR2 | 4920 | GUUUGCAUGUGCCGGAAUA |
|  |  | CGACAGACACUGGCUACUA |
|  |  | GCAACCGGACCAUUUAUGU |
|  |  | GCUCAGGCAUGGAUUACAG |
| DVL1 | 1855 | GACCAAGGCCUAUACAGUG |
|  |  | GCGAGUUCUUCGUGGACAU |
|  |  | GAUCACACGGCACCGAGUG |
|  |  | GGGGAUCUCUGCAGCAAUC |
| DVL2 | 1856 | GCUCAAAGCAGGCCUGAUC |
|  |  | GGGAGACGAAGGUGAUUUA |
|  |  | CGCUAAACAUGGAGAAGUA |
|  |  | CCACAAUGUCUCUCAAUAU |
